# Patient-based benefit-risk assessment of medicines: development, refinement, and validation of a content search strategy to retrieve relevant studies

**DOI:** 10.5195/jmla.2022.1306

**Published:** 2022-04-01

**Authors:** Hiba El Masri, Treasure M. McGuire, Christine Dalais, Mieke van Driel, Helen Benham, Samantha A. Hollingworth

**Affiliations:** 1 h.elmasri@uqconnect.edu.au, PhD Candidate, School of Pharmacy, The University of Queensland, Woolloongabba, QLD, Australia; 2 t.mcguire@uq.edu.au, Faculty of Health Sciences and Medicine, Bond University, Robina, QLD, Australia, Mater Pharmacy, Mater Health, Raymond Tce, South Brisbane, QLD, Australia; 3 c.dalais@library.uq.edu.au, University Library, The University of Queensland, Brisbane, QLD, Australia; 4 m.vandriel@uq.edu.au, Primary Care Clinical Unit, Faculty of Medicine, The University of Queensland, Brisbane, QLD, Australia; 5 h.benham@uq.edu.au, Department of Rheumatology, Princess Alexandra Hospital, Brisbane, QLD, Australia; 6 s.hollingworth@uq.edu.au, School of Pharmacy, The University of Queensland, Brisbane, QLD, Australia

**Keywords:** patient-based benefit-risk assessment, benefit-risk assessment, attribute development, patient preference, prescription drug, risk assessment/methods, databases, bibliographic, information storage and retrieval/methods, information storage and retrieval/standards, Medical Subject Headings, terminology as topic, reproducibility of results

## Abstract

**Introduction:**

Poor indexing and inconsistent use of terms and keywords may prevent efficient retrieval of studies on the patient-based benefit-risk assessment (BRA) of medicines. We aimed to develop and validate an objectively derived content search strategy containing generic search terms that can be adapted for any search for evidence on patient-based BRA of medicines for any therapeutic area.

**Methods:**

We used a robust multistep process to develop and validate the content search strategy: (1) we developed a bank of search terms derived from screening studies on patient-based BRA of medicines in various therapeutic areas, (2) we refined the proposed content search strategy through an iterative process of testing sensitivity and precision of search terms, and (3) we validated the final search strategy in PubMed by firstly using multiple sclerosis as a case condition and secondly computing its relative performance versus a published systematic review on patient-based BRA of medicines in rheumatoid arthritis.

**Results:**

We conceptualized a final search strategy to retrieve studies on patient-based BRA containing generic search terms grouped into two domains, namely the patient and the BRA of medicines (sensitivity 84%, specificity 99.4%, precision 20.7%). The relative performance of the content search strategy was 85.7% compared with a search from a published systematic review of patient preferences in the treatment of rheumatoid arthritis. We also developed a more extended filter, with a relative performance of 93.3% when compared with a search from a published systematic review of patient preferences in lung cancer.

## INTRODUCTION

Medicines are used for a known therapeutic benefit including cure, delaying disease progression, relieving symptoms, or preventing comorbidities. Every medicine also carries a risk of side effects, from minor to severe. A thorough understanding of both benefits and risks of every pharmacotherapeutic intervention should underpin the effective medicine management cycle of prescribing, dispensing, administering, and monitoring of effect [1]. Balancing benefits and risks is also a key step in the decision-making process of regulatory authorities and in developing guidelines by professional societies [2].

The methodical and regular review of the efficacy and safety parameters of a medicine is called a benefit-risk assessment or analysis (BRA) or benefit-risk ratio evaluation. BRA is primarily an exercise that balances two dimensions: the dimension of benefit that includes not only therapeutic efficacy but also improvement of quality of life, and the dimension of risks that consists of the safety profile of the given medicine and the potential risk of unintended adverse events anticipated on the basis of the mechanism of action [3]. The dimension of cost could also be embedded in this analysis [4].

Despite the use of quantitative and structured approaches to evaluate the benefits and risks of medicines, the BRA remains heavily influenced by the value judgments of clinical experts [2]. This professional BRA judgment does not necessarily correlate with patients' evaluations. In fact, there is growing evidence that the trade-offs between benefits and risks made by patients differ significantly from clinical experts [5]. Furthermore, patients with chronic conditions cautiously and deliberately reassess the benefits and risks of their treatments at multiple milestones in their disease journey and may overemphasize the potential risks of their current medicines or overestimate the benefits of new treatments [6]. The concept of a patient-based BRA of medicines has recently emerged and is attracting the attention of regulatory authorities who acknowledge the importance of incorporating patients' perspectives into their decision-making processes [7, 8].

There has been a steady increase in studies eliciting patients' preferences and perspectives on their medicines [9, 10]. The growing evidence about patients' perspectives in general—and patient-based BRA in particular—is likely to inform clinical and regulatory decision-making [11]. There are barriers, however, to comprehensive and efficient searching for this evidence, including poor indexing and the inconsistent use of terms to denote either patient preferences or patient-based BRA [12]. Moreover, the capacity of most methods used to accurately elicit patients' perspectives depends on the researcher selecting a medicine's attributes and the patient's understanding the proposed choices [13]. A robust search strategy with predictable performance parameters is needed to rigorously generate and develop BRA attributes.

While some search strategies have been proposed to identify the literature on patients' knowledge, views, and preferences about their health and health care [14–16], we could not retrieve any search strategy to selectively identify how patients balance the benefits and risks of their medicines. The former search strategies produce a wide search yield, containing studies on patients' perspectives in various areas such as information and knowledge needs, communication and social support, appraisal of symptom severity, comorbidity management, hospitalization, and prevention and screening tests. We aimed to create and validate a targeted, objectively derived content search strategy for patient-focused BRA of medicines that would detect studies on patients' perspectives on medicines' attributes, assessments of adverse event severity, the importance of avoiding side effects, and other treatment characteristics such as routes of administration, frequency of treatment, inconvenience caused by scheduled treatment, and costs. The measures of this validated and objectively derived search strategy would be calculated. Search strategy performance would be predictable, unlike subjectively derived strategies that are mainly based on the authors' expertise, with their methodologies not consistently reproducible. The proposed content search strategy would contain generic search terms, rather than terms specific to a given therapeutic area, allowing it to be used as a search filter for patient-based BRA of medicines in any therapeutic area.

## METHODS

**Figure 1:**
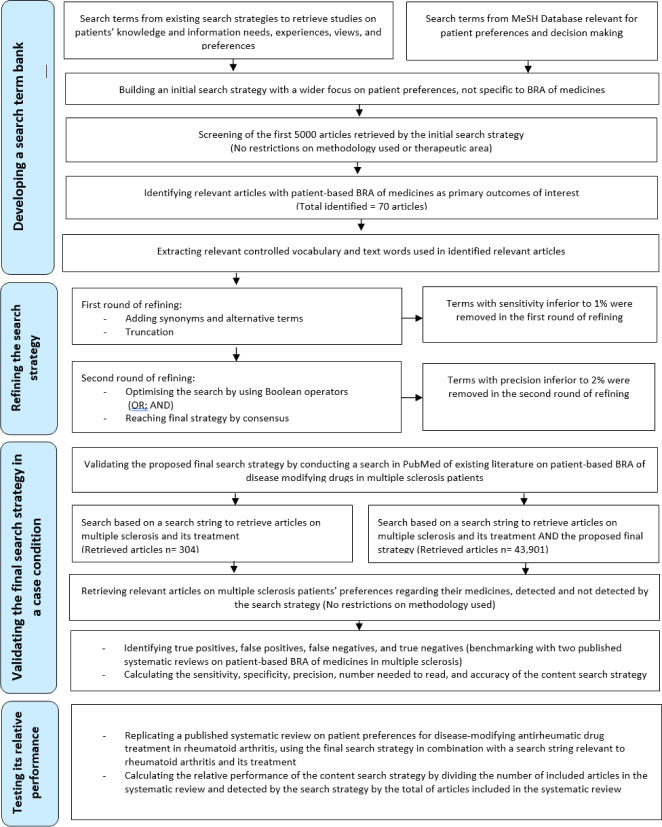
Flow diagram depicting the iterative process of developing a search term bank, refining the search strategy, and then validating the final strategy

We developed a content search strategy to retrieve studies on patient-based BRA of medicines using an iterative process: (1) developing a search term bank, (2) refining the search strategy, and (3) validating the final strategy (Figure 1). This content search strategy was constructed and validated in PubMed. PubMed is a free resource supporting the search and retrieval of biomedical and life sciences literature and is one of the most commonly used medical databases [15].

### Developing a bank of candidate search terms

We compiled a list of search terms (Medical Subject Heading [MeSH] terms, other indexed terms in title or abstract, and free text) from two sources: (1) existing search strategies used to retrieve studies on patients' knowledge and information needs, experiences, views, and preferences [14–16] and (2) the MeSH database in PubMed [17]. The MeSH thesaurus is a controlled and hierarchically organized vocabulary established by the National Library of Medicine for indexing and searching biomedical and health-related information [17].

We combined these terms into a subjectively derived search filter, or hedge, that was developed based on several sources: the authors' subject knowledge, expertise of our librarian searcher, existing search strategies, the MeSH database, and a thesaurus (Appendix 1) [18]. We ran an initial search (using OR) in PubMed in July 2020. Although the patient was at the core of our initial search strategy, we expected it would yield a large number of articles under the broad scope of patients' preferences in all aspects of health and illness, including preferences related to the attributes of their medicines. The initial search yielded 790,674 articles. For practical purposes, we manually screened the first 5,000 articles (by order of appearance) retrieved by this combined list of search terms without limiting our search by the nature of the medical or health condition, language, or date of publication. We also used Best Match rather than chronological order as the active filter in PubMed to enable relevant older citations to be retrieved. However, our a priori decision was to continue beyond this citation limit if insufficient relevant papers were retrieved or search term saturation was not achieved. We first screened at the level of title and abstract, to determine which articles discussed patient-based BRA of medicines.

We identified seventy relevant papers on patients' preferences for treatment attributes in different therapeutic areas, published in many journals (Appendix 2). The studies were deemed relevant if they elicited patient-based BRA of medicines using quantitative and or qualitative approaches. The identified studies covered many therapeutic categories and medical conditions, mostly chronic in nature (e.g., cardiology, endocrinology, neurology, nephrology, dermatology, immunology, rheumatology, gastroenterology, oncology, and other therapeutic areas). We extracted controlled vocabulary (i.e. author-designated keywords and MeSH terms plus subheadings) and free text words from abstracts and full texts of the seventy identified papers on patient-based BRA of medicines (Appendix 2). The resulting collection of terms, including all terms from the hedge, formed our bank of candidate search terms.

### Refining the search strategy

In developing methodological search strategies, experts define the gold standard (also called reference standard or reference set) as a set of relevant records against which the search strategy is tested and validated to determine its performance parameters [18]. Through an iterative process and two rounds of discussions, two authors (HM and SH) screened and tested the bank of candidate search terms to optimize the effectiveness and efficiency of the search without making the search yield too narrow. The analytical approach to refine the terms to be included in the content search strategy was based on frequency of occurrence, often used by information specialists and experts to develop and validate objectively derived search strategies [19–21]. We set the minimum threshold of frequency of occurrence for an individual term to be included by multiple testing of various combinations to obtain optimal performance measures, in particular sensitivity, precision, and specificity. The sensitivity or recall rate is defined as the number of relevant records in the gold standard retrieved by the search strategy as a proportion of the total number of records in the gold standard. The precision or positive predictive value (PPV) is the number of relevant records retrieved by the search strategy as a proportion of the total number of records retrieved. The specificity is the number of records that are not relevant and are not retrieved by the search strategy as a proportion of the total number of nonrelevant records [18]. Equations used to calculate performance parameters are given in Table 1.

**Table 1:** Formulas used to calculate the sensitivity, specificity, precision, accuracy, and NNR of the content search strategy

Search syntax applied	Relevant articles	Nonrelevant articles	Total
Content search strategy + (Search A) Search strategy combined (using AND) with the MS search string*	a True positives	b False positives	a + b
Content search strategy – (Search B) Use of the MS search string* alone	c False negatives	d True negatives	c + d
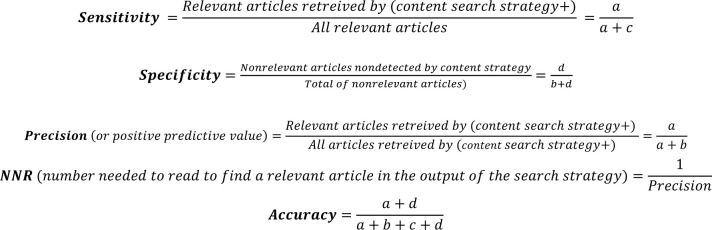			

* Search string used to retrieve articles on multiple sclerosis and its treatment: (((multiple sclerosis) OR (multiple sclerosis[MeSH Terms])) OR (Multiple Sclerosis / therapy[MeSH Terms])) OR (Multiple Sclerosis, Relapsing-Remitting / drug therapy[MeSH Terms])

We performed our term frequency analysis in two rounds: in the first round we retained the terms with sensitivity higher than 1%, and in the second round we retained the terms with precision greater than 2%. We set the minimum thresholds of frequency of occurrence by multiple testing of various combinations to obtain optimal performance. We aimed for a minimum of 80% for sensitivity and 20% for precision. The balance between sensitivity and precision was determined with the thresholds 1% and 2%, with consensus among all authors. For an extended search strategy, we aimed for a minimum of 90% for sensitivity at the cost of any precision. In the extended search strategy, we included all the terms that surpassed the 1% sensitivity threshold (Appendix 3).

In this refining exercise, we chose type 2 diabetes mellitus (T2DM) as a case condition to test the performance of each search term. We chose T2DM for four reasons: (1) T2DM is highly prevalent and associated with significant excess risk in cardiovascular morbidity and mortality [22], (2) treatment can be with oral or parenteral glucose-lowering agents, with various device models available, (3) newer oral-glucose-lowering agents are suggested to have better cardiovascular effects than long-established glucose-lowering agents [23, 24], and (4) clinical recommendations stress the importance of a personalized and patient-centered treatment approach [25]. There are many studies exploring patient-based preferences of medicines for treating T2DM [26, 27], which makes it a suitable area to test the performance of our compiled search terms.

For terms relevant to T2DM and its management, we used this search string:

Diabetes Mellitus / therapy[MeSH] OR Diabetes Mellitus, Type 2 / drug therapy[MeSH] OR Diabetes Mellitus, Type 2 / therapy[MeSH] OR Hypoglycemic Agents / administration & dosage[MeSH] OR Hypoglycemic Agents / therapeutic use[MeSH] OR Hypoglycemic Agents / adverse effects[MeSH] OR Hypoglycemia / prevention & control[MeSH] OR Blood Glucose / drug effects[MeSH] OR Glucagon-Like Peptides / therapeutic use[MeSH] OR Insulin / therapeutic use[MeSH] OR Insulin / administration & dosage[MeSH].

To determine a gold standard reference set for performance measurements in this refining exercise, we combined—in PubMed—all candidate search terms (using OR) with the T2DM search string (given above) using AND. We screened on title and abstract level all retrieved records for relevant studies on patient-based BRA of medicines in T2DM; this constituted our gold standard reference set. We then tested in PubMed, one by one, each of the terms in our bank by combining them (using AND) with the T2DM search string.

We removed redundant terms that retrieved irrelevant studies. We added synonyms and alternative terms to the search terms. We applied truncation techniques to selected search terms—in other words, a deliberate shortening of a search term by adding a wildcard character (e.g., *) to retrieve other variants of the word due to differences in descriptions, language, or spelling [28, 29]. Terms with a sensitivity value higher than 1% were retained in the first round of refining. We further optimized the search by using Boolean operators (OR; AND) [30] to combine the search terms from the developed bank and reflect the two components of the patient-focused BRA concept (i.e., the patient and the balance between benefit and risk including all attributes of the medicines). In the second round we retained terms with satisfactory sensitivity (higher than 1%) and precision value greater than 2%. We reached the proposed final version of the search strategy by consensus among authors. We also put all the terms retained in the first round (sensitivity higher than 1%) into an extended content search strategy (Appendix 3).

### Validating the search strategy

We validated the approach at two levels. First, we validated the search strategy in a case condition and computed its performance parameters. Second, we tested its relative performance by comparing it to the search approach in a published systematic review.

#### Validating the search strategy in a case condition

We validated the proposed final search strategy in PubMed by conducting a search of existing literature on patient-based BRA of disease-modifying drugs used to treat multiple sclerosis (MS) as a case condition. The choice of MS was based on (1) factors related to the disease, where patients experience significant pain and disability during their disease journey and may face multiple relapses and remissions, and (2) factors related to the newer, more-effective treatment options, which are also associated with increased risks of severe adverse events [31]. These two factors make MS patients' perceptions of benefits and risks of their medicines crucial in the shared decision-making process of managing their condition [32, 33]. The complex risk-benefit profiles of MS treatment options and the greater involvement of patients in their treatment decisions than in other diseases have made MS a rich area to explore patient-based BRA of medicines [34].

We used this search string:

Multiple sclerosis OR Multiple Sclerosis [MeSH Terms] OR Multiple Sclerosis / therapy [MeSH Terms] OR Multiple Sclerosis, Relapsing-Remitting / drug therapy [MeSH Terms].

We ran the search in PubMed in August 2020, with (using AND) and without the proposed search strategy and applied a ten-year filter for the publication date (from August 2010 to August 2020). Search A was based on the search string of MS combined with the developed search strategy (using AND) and search B was based on the search string of MS alone. We screened all records in search A on title and abstract level. We considered a study as relevant if the outcomes of interest included the preferences of MS (any subtype) patients toward their medicines, ideally specified according to clinical or convenience attributes describing various treatment scenarios. We did not exclude any study based on the methodology (quantitative or qualitative, empirical or review). We categorized articles as either relevant (true positives) or not relevant (false positives).

We then ran the search for articles on MS and its treatment, using the above search string alone, with the same ten-year filter for the publication dates (search B). We screened all records in search B on title and abstract level. The false negatives were considered the relevant articles that were only detected without adding the search strategy (detected only by applying the string to retrieve articles on MS and its treatment—search B). These were the relevant articles that the search strategy failed to detect. The remaining articles retrieved without the use of the search strategy were the true negatives. Because there is no established standard to ensure identifying all relevant articles on MS and its treatment [15]—thus identifying all false negatives—we compared our list of “relevant detected” and “relevant not detected” articles to those included in two published systematic reviews on patients' preferences for risks and benefits of disease-modifying drugs in MS [34, 35]. All articles listed in the two reviews and published on or after August 2010 (Appendix 4) were already included in our list of “relevant detected” and “relevant not detected” articles. Assuming that the pool of identified papers is as near as possible to the total number of relevant papers, we computed the sensitivity, specificity, precision, and number needed to read (NNR) of the content search strategy (Table 1) [36, 37].

#### Testing the relative performance of the search strategy by replicating a published systematic review

Systematic reviews aim to comprehensively locate and appraise research on a particular question, using structured and replicable procedures at each step of the process [38]. The approach and corresponding search strategies adopted in published systematic reviews to identify literature on patient-based BRA of medicines are considered the gold standard in this field [39]. As there are no established criteria to assess risk of bias or the methodological quality of patient preference studies [40], we adopted the checklist constructed by Eiring and colleagues [41] to choose quality systematic reviews for chronic diseases of interest. The checklist consisted of thirty-one quality criteria within five main domains: (1) external validity of the study, (2) quality of construct representation, (3) minimization of the risk of construct-irrelevant variance, (4) quality of reporting and analysis, and (5) other aspects that may strengthen or weaken the study.

We compared the performance of our developed content search strategies to the results of a published systematic review on patient preferences for disease-modifying antirheumatic drug (DMARD) treatment in rheumatoid arthritis (RA) [42]. In this systematic review, thirty-one studies had an overall quality ranging from medium to high, and four had a low overall quality (Appendix 5).

We combined the developed strategy (using AND) with the following search terms:

Rheumatoid arthritis OR RA OR rheumatic diseases OR disease-modifying antirheumatic drugs OR DMARDs OR antirheumatic agents OR Arthritis, Rheumatoid / drug therapy [MeSH] OR Arthritis, Rheumatoid / therapy [MeSH] OR Antirheumatic Agents / therapeutic use [MeSH] OR Biological Products / therapeutic use [MeSH].

We ran the search in PubMed in December 2020. We calculated the relative performance of the content search strategy in PubMed by dividing the number of articles included in the systematic review and detected by the search strategy by the total number of articles included in the systematic review and cited in PubMed [14].

#### Testing the relative performance of the extended search strategy by replicating a published systematic review

We compared the performance of the extended content search strategy to the results of a published systematic review on patient preferences for lung cancer treatment [43]. In this systematic review, fourteen studies had an overall quality ranging from medium to high, and one study had a low overall quality (Appendix 3).

We combined the extended content search strategy (using AND) with the following search terms:

Lung Neoplasm [MeSH] OR Antineoplastic Agents [MeSH] OR lung cancer [tiab]

We ran the search in PubMed in July 2021. We calculated the relative performance of the content search strategy in PubMed by dividing the number of articles included in the systematic review and detected by the extended search strategy by the total number of articles included in the systematic review.

## RESULTS

### Developing a bank of search terms and refining the search strategy

We conceptualized a final search strategy to retrieve studies on patient-based BRA after a series of refining rounds. The search terms are grouped into two domains: (1) domain of the patient and (2) domain of the BRA of medicines (Table 2). The reproducible search strategy can also be found in Appendix 6. Terms within each domain were combined using the Boolean operator OR, and the two domains were combined using the operator AND. Several search entries were directly sourced from the MeSH database, such as Patient Preference, Choice Behavior, Benefit Risk Assessment, and Risk Assessment. There were no specific entries corresponding to patient-based risk assessment or patient-based BRA below the corresponding terms or other related terms in the MeSH hierarchy. Most search terms included in the BRA domain were in the form of free text extracted from our review of selected studies of patients' understanding and preferences for risks and benefits of their medicines. In contrast, the terms compiled in the patient domain derived mostly from MeSH terms and subheadings in existing search strategies. In the initial search, these strategies identified articles with relatively high sensitivity and acceptable precision with a focus on patients' views and perceptions of all aspects of their disease and health management.

**Table 2:** Content search strategy of patient-based benefit-risk assessment

Domain of the patient	Domain of benefit-risk assessment
Patient Preference [MeSH] Patient Preference / psychology [MeSH Subheading] Patient Preference / statistics & numerical data [MeSH Subheading] Patient Preference* [tiab] Patients preference* [tiab] Patient perception[tiab] Stated preference* [tiab] Treatment preference [tiab] Preference [tiab] Perspective [tiab] Choice Behavior [MeSH] Decision Making[MeSH] Health Knowledge, Attitudes, Practice[MeSH]	Attribute* [tiab] Benefit* [tiab] Benefit-risk [All fields] Risk tolerance [All fields] Trade-off* [All fields] Tradeoff* [All fields] Efficacy [tiab] Safety [tiab] Side effect* [tiab] Adverse event* [tiab] Adverse reaction* [tiab] Effectiveness [tiab] Frequency [tiab] Accepta* [tiab] Maximum acceptable risk [All fields] Minimum acceptable efficacy [All fields] Preferred treatment option [tiab] Patient-reported outcome* [tiab] Relative importance [tiab] Most preferred [tiab] Least preferred [tiab] Willingness [tiab] Risk Assessment [MeSH] Benefit risk assessment [MeSH] Drug-related side effects and adverse reactions/psychology [MeSH]

We further refined the search strategy when we added the concept of medicine or treatment to the approach. Many retrieved studies discussed patients' participation in physical activity and screening activities rather than eliciting patients' preferences about their medicines. Most studies were retrieved using the term “participation,” so we deemed terms related to participation (patient's participation, user's participation) as redundant (sensitivity of each term below 1%). Similarly, most of the studies retrieved using the term “patient satisfaction” (not treatment satisfaction) focused on patients' assessment of health services, programs, and use of devices (sensitivity below 1%), so we removed this search term.

We included in our initial bank of search terms as many entries that identified articles relevant to patients' preferences and choice behavior as possible (e.g., user, user's, users, consumers, individuals, based, focused, centered), given the inconsistencies in the nomenclature and indexing about patients' preferences and perspectives on medicines. We noticed during the refinement stage that the term “patient” was more often used in studies examining the BRA of medicines, but the terms “user” and “consumer” were more often used in studies about devices and services. We excluded the latter terms in the final search strategy (sensitivity of each term below 1%). We used truncation on selected free-text entries to reduce the number of terms (e.g., accepta* covered acceptance, acceptability, and acceptable). The simultaneous use of both the MeSH term and the corresponding free text yielded more results than either alone. This was the rationale for the final strategy containing double entries to be searched in “All fields OR in MeSH.”

The refining component was based on the search terms identified in the screening of seventy studies focused on patient-based BRA of medicines (Appendix 2) from various therapeutic areas. We derived search terms from fifty-seven different journals with different indexing requirements and use of terms. The refining component comprised an iterative process of testing each term for sensitivity and precision. We used T2DM as a case condition. We included terms in the final strategy if they had acceptable sensitivity (more than 1% in the first round) and precision (higher than 2% in the second round). The individual precision varied widely across included search terms as well as the number of total articles retrieved per term. For example, stated preference*[tiab] had the highest precision (70.0%) but with seven relevant articles out of ten retrieved, whereas Patient Preference*[MeSH] had the second highest (33.3%) with 67 relevant articles out of 201 retrieved. Tradeoff*[All fields], willingness[tiab], and Choice Behavior[MeSH] also had high individual precision: 24.4% (20 relevant articles of 82 retrieved), 15.2% (44 relevant articles of 289 retrieved), and 9.7% (20 relevant articles of 207 retrieved), respectively. The rest of the included terms had lower precisions, with substantially higher numbers of articles retrieved like attribute*[tiab] (4.6%, 46 relevant articles of 998 retrieved); Health Knowledge, Attitudes, Practice [MeSH] (3.0%, 57 relevant articles of 1,875 retrieved); and Benefit Risk Assessment [MeSH] (2.0%, 46 relevant articles of 2,266). The combination of terms with Boolean operators enabled us to optimize the performance of the overall content search strategy.

While refining the compiled search terms, we found selected entries (free text and controlled vocabulary) with passable sensitivity (more than 1%) but very low precision (less than 2%). These entries, if used together, increased the sensitivity of the search strategy but decreased the precision. These search terms were in both domains: (a) the patient (e.g., treatment satisfaction, perception, Patient Acceptance of Health Care [MeSH]), and (b) the BRA of medicines (e.g., discontinuation, medication belief*, patient-relevant benefit, Risk [MeSH], Treatment Outcome [MeSH]). We built an extended search strategy containing the final refined strategy combined with all these additional terms (Appendix 3).

### Validation of the content search strategy

#### Validating the search strategy in MS as a case condition

We validated the final search strategy by combining it (using AND, Filter applied: in the last 10 years) with the relevant search terms for MS in PubMed. Search A returned 304 results: 63 relevant (true positives) and 241 not relevant to patient-based BRA of DMARDs (false positives). A search using only the relevant search terms for MS (Filter applied: in the last 10 years—search B) returned 43,901 hits including 75 relevant (which included 12 false negatives) and 43,825 true negatives. False negatives are the relevant articles that the search strategy failed to detect. All relevant articles (true positives and false negatives) are listed with their corresponding keywords and MeSH terms in Table 3. The sensitivity of the content search strategy was 84.0%, the specificity was 99.4%, and the accuracy was 99.4%. The PPV was 20.7% and the NNR was 4.8 studies (Table 4).

**Table 3:** Relevant articles on multiple sclerosis patients' preferences regarding their medicines detected (RD) and not detected (RND) by the search strategy with corresponding keywords and MeSH terms

Article	RD	RND	Keywords / MeSH terms	
			Patient-focused	Benefit-risk assessment of medicines
Abolfazli et al. {50]		X	Perspectives Attitude	Self-injection
Arenson [51]	X		Decision Making* [MeSH] Health Knowledge, Attitudes, Practice*[MeSH]	Risk Assessment [MeSH]
Arroyo et al. [52]	X		Patient preferences Decision Making* [MeSH] Patient Preference*[MeSH]	Attributes Risk-benefit
Barone et al. [53]	X		Patient perceptions Patient satisfaction	Attributes
Bauer et al. [54]	X		Patient preferences	Dosing regimen Efficacy Safety Side effects Treatment preferences
Bayas et al. [55]	X		Patient preferences Treatment decision process	Potential side effects
Beckmann et al. [56]		X	Patient Satisfaction [MeSH]	Patient-relevant benefits Treatment Outcome*[MeSH]
Bichuetti et al. [57]	X		Perception*[MeSH] Health Knowledge, Attitudes, Practice [MeSH]	Risk Assessment [MeSH] Risk-Taking*[MeSH]
Boeru et al. [58]	X		Patient preference	Adverse events Severity of adverse events
Bottomley et al. [59]	X		Patient preference Choice Behavior* [MeSH] Decision Making [MeSH] Patient Preference / psychology* [MeSH]	Attributes Most/least preferred options Drug-Related Side Effects and Adverse Reactions / epidemiology [MeSH]
Brown et al. [60]	X		Patient engagement Patient Participation [MeSH] Decision Making*[MeSH]	Efficacy
Bruce et al. [61]	X		Decision Making / physiology* [MeSH]	Side effects Risk and benefit probabilities Treatment Outcome [MeSH]
Bruce et al. [62]	X		Decision Making*[MeSH] Health Knowledge, Attitudes, Practice*[MeSH]	Risks Benefits
Bruce et al. [63]	X		Willing Willingness Medication beliefs	Risk-benefit Trade-off Risk Assessment
Carlin et al. [64]	X		Patient Preference* [MeSH] Choice Behavior* [MeSH] Attitude to Health* [MeSH]	Attributes Side effects Drug-Related Side Effects and Adverse Reactions [MeSH] Risk [MeSH]
Ceuninck van Capelle et al. [65]		X	Patient perspectives Decision Making*[MeSH] Patient Participation*[MeSH]	Prevent relapses Prevent disease progression Disease Progression [MeSH]
Cocco et al. [32]	X		Engagement Share decision-making Participation preference Decision Making [MeSH]	Perception of risk Benefits Risks Risk [MeSH]
Col et al. [66]	X		Decision Making*[MeSH] Patient Preference [MeSH]	Attribute Outcome
Col et al. [67]	X		Patient preference Shared decision making	Attributes Preference domains
de Seze et al. [68]	X		Perception	Treatment outcome
Eskyte et al. [33]	X		Treatment decisions Perspective of people Decision Making*[MeSH] Patient Participation [MeSH]	Treatment Outcome [MeSH]
Fernández et al. [69]	X		Stated preference Patient Satisfaction* [MeSH]	Administration routes Effectiveness
Fox et al. [70]	X		Decision Making*[MeSH] Health Knowledge, Attitudes, Practice [MeSH]	Tolerance Risk acceptance Risk [MeSH]
Frost et al. [71]	X		Patient preference Willingness-to-pay Patient Preference / statistics & numerical data*[MeSH]	Attributes Relative preferences
Garcia-Dominguez [72]	X		Patient preferences	Attribute The most important factor Maximum acceptable risk
Glanz et al. [73]		X	Participants Individuals	Risk attitude Risk perception Tolerance for risk
Goodwin et al. [74]	X		Patient preferences Preference elicitation Patient Preference*[MeSH]	Trade-off
Heesen et al. [75]	X		Choice Behavior [MeSH]	Benefit-risk Risk acceptance Risk Assessment [MeSH]
Heesen et al. [76]	X		Perception Willing Health Knowledge, Attitudes, Practice*[MeSH] Perception [MeSH]	Accept higher risks Risks and benefits Risk [MeSH]
Hincapie et al. [77]	X		Decision Making [MeSH] Patient Preference / economics*[MeSH]	Attributes Adverse effects Efficacy Mode of administration
Hofmann et al. [78]	X		Perception Health Knowledge, Attitudes, Practice* [MeSH]	Treatment benefits and risks Risk Risk awareness Risk estimation Risk [MeSH]
Jarmolowicz et al. [79]	X		Decision Making*[MeSH]	Probabilistic benefit Side effect severity Treatment Outcome [MeSH]
Jarmolowicz et al. [80]	X		Choice Behavior*[MeSH]	Benefits Cost/benefit ratio
Jarmolowicz et al. [81]	X		Choice Behavior [MeSH]	Side-effect probabilities Side-effect severities Drug-Related Side Effects and Adverse Reactions / epidemiology*[MeSH]
Köpke et al [82]	X		Preferences Decision Making [MeSH] Health Knowledge, Attitudes, Practice*[MeSH]	Effectiveness
Kremer et al. [83]	X		Preferences of patients Choice Behavior [MeSH] Decision Making* [MeSH]	Attributes Severity of side effects Most and least important attributes
Kremer et al. [84]	X		Patient Preference*[MeSH] Decision Making*[MeSH]	Attributes Relative importance
Lee Mortensen et al. [85]	X		Patient preferences	Treatment side effects Mode of administration Treatment preferences
Lin et al. [86]	X		Patient Preference / statistics & numerical data*[MeSH]	Relative importance
Lizán et al. [87]	X		Patients' preference Patients' needs	Attributes Treatment preferences
Lynd et al. [88]	X		Patient perspective Patient preference	Effectiveness and side effects Risks Benefits
Lynd et al. [89]	X		Patient preference	Attribute The most important attributes Risk to benefit tradeoff
Mansfield et al. [90]	X		Patient preferences Treatment decisions	Attributes
McDonnell et al. [91]		X	Respondents' attitude	Risk tolerance Drug Tolerance [MeSH] Risk Management [MeSH]
McGinley et al. [92]		X	Patient engagement Opinion of individuals Decision-making	Discontinuation
Mendel et al. [93]	X		Patient Participation [MeSH] Patient Preference*[MeSH]	Preferred treatment option
Miller et al. [94]		X	Patients' experience	Increased risk Benefits
Poulos et al. [95]	X		Stated preference Choice Behavior [MeSH] Patient Preference / psychology* [MeSH]	Attributes Minimum acceptable efficacy
Poulos et al. [96]	X		Patient preference Stated preference	Attributes Relative importance
Poulos et al. [97]	X		Patient Preference / psychology [MeSH] Patient Preference / statistics & numerical data* [MeSH] Choice Behavior [MeSH]	Attributes Relative importance
Poulos et al. [98]	X		Patient preferences	Attributes The most important attributes Treatment preferences Severe side-effect risks
Rahimi et al. [99]	X		Choice Behavior* [MeSH] Patient Preference*[MeSH]	Attributes Efficacy Side effects Utility
Rath et al. [100]	X		Health Knowledge, Attitudes, Practice*[MeSH]	Treatment risks Risk Assessment [MeSH] Treatment Outcome [MeSH]
Reen et al. [34]	X		Patient Preference* [MeSH] Decision Making*[MeSH]	Risks and benefits of treatments Risk Assessment [MeSH]
Rosato et al [101]	X		Patient preferences	Attributes
Salamonsen [102]	X		Perception [MeSH] Health Knowledge, Attitudes, Practice [MeSH]	Risk perception Severe adverse effects Risk* [MeSH]
Salter et al. [103]	X		Patient perspectives Satisfaction	Effectiveness
Sempere et al. [104]	X		Patient preferences Decision-making	Attributes Most preferred Least preferred Routes and schedule of administration
Shingler et al. [105]	X		Choice Behavior* [MeSH] Patient Preference / statistics & numerical data* [MeSH]	Attributes
Syed et al [106]		X	Patient expectations Experience	Treatment discontinuation
Thach et al. [107]		X	Treatment satisfaction	Medication beliefs
Thakur et al. [108]	X		Patients' perceptions Patient preference	Attributes
Tourbah et al. [109]	X		Patient preference	Tolerability Acceptability Effectiveness Adverse events
Tur [110]		X	Perception Perception / drug effects* [MeSH]	Risk acceptance Risk Factors [MeSH]
Turčáni et al. [111]		X	Treatment satisfaction Patient satisfaction	Effectiveness
Utz et al. [112]	X		Patient preference	Attribute Route of administration Treatment frequency
Visser et al. [113]	X		Patient preferences	Attributes Relative importance
Visser et al. [114]	X		Patient Preference*[MeSH]	Side effects
Volpicelli Leonard et al. [115]	X		Patient perception	Effectiveness Treatment satisfaction
Webb et al. [35]	X		Stated preference Choice Behavior [MeSH] Decision Making [MeSH] Patient Preference*[MeSH]	Attributes
Wicks et al. [116]	X		Patient preference Decision making	Attribute Relative importance
Wilkie et al. [117]		X	Decision-making	Offered treatment Dissatisfaction
Wilson et al. [118]	X		Decision Making / physiology* (Mesh) Patient Preference / psychology* [MeSH]	Risk–benefit Trade-offs Utility Attributes Risk Assessment [MeSH]
Wilson et al. [119]	X		Patient preference	Attributes Risks and benefits of treatment
Zimmer et al [120]	X		Perception Patient satisfaction Health Knowledge, Attitudes, Practice [MeSH] Perception [MeSH] Patient Satisfaction [MeSH]	Efficacy Safety Treatment Outcome [MeSH]

RD: relevant detected; RND: relevant not detected

**Table 4:** Validation of the content search strategy and performance calculation

Search syntax applied	Relevant articles	Nonrelevant articles
Content search strategy + (Search A) Search strategy combined (using AND) with the MS search string*	62	241
Content search strategy – (Search B) Use of the MS search string* alone	13	43,825
*Sensitivity* = 84% *Specificity* = 99.4% *Accuracy* = 99.4% *Precision* = 20.7% *NNR* = 4.8 *Accuracy* = 99.4%		

* Search string used to retrieve articles on multiple sclerosis and its treatment: (((multiple sclerosis) OR (multiple sclerosis[MeSH Terms])) OR (Multiple Sclerosis / therapy*[MeSH Terms])) OR (Multiple Sclerosis, Relapsing-Remitting / drug therapy[MeSH Terms])

#### Relative performance of the content search strategy

The systematic review on patient preferences for DMARD treatment in RA included 36 unique studies [42]. We used 35 studies to benchmark the output of the content search strategy as one study was not cited in PubMed [44]. Our search combining the developed content search strategy with the search syntax relevant to RA yielded 30 of the 35 studies: an 87.5% relative performance of the content search strategy. All articles in the systematic review (detected and not detected by the content search strategy) are listed with their corresponding keywords and MeSH terms in Appendix 5.

#### Relative performance of the extended search strategy

The systematic review on patient preferences for lung cancer treatment included fifteen unique studies [43]. We used all the included studies to benchmark the output of the extended search strategy, as all were listed in PubMed. The search combining the extended content search strategy with the search syntax relevant to lung neoplasm detected fourteen of the fifteen studies. This equates to 93.3% relative performance for the extended filter. All articles in the systematic review (detected and not detected by the extended content search strategy) are listed with their corresponding keywords and MeSH terms in Appendix 3 (Table 2). The reproducible extended search strategy can also be found in Appendix 6.

## DISCUSSION

We developed a high-performance, objectively derived search strategy for patient-focused BRA of medicines. This search filter had high sensitivity for studies about patients' preferences and perspectives on the benefits and risks of their medicines, with excellent specificity and accuracy, and acceptable precision. Our proposed content search strategy was more targeted to retrieve studies on how patients perceive the effectiveness of their medicines and whether the potential benefits outweigh the harms when compared with another search filter designed to identify existing literature on patients' knowledge, views, and values [15]. The main advantage of our search strategy is that it has been empirically developed and validated based on clearly defined, pragmatic, and reproducible methods [20]. The use of such strategies could minimize the time, biases, and potential obstacles associated with those subjectively derived, such as the need for multiple search queries to make the search sufficiently wide and the subsequent need to restrict when the search yield is too broad [20, 45].

Patient-centered care encompasses shared decision-making, support for self-management, patient information, patient empowerment, care planning, the integration of medical and nonmedical care, and good communication between clinicians and patients [46]. It is also important to understand patients' preferences in all aspects of their disease management. It is therefore important to efficiently collect evidence on the priorities and perspectives of patients regarding their medicines for prescribing, developing clinical guidelines, or making decisions in health technology assessment [39, 47].

We detected the search terms mostly used in studies relevant to patient-focused BRA of medicines by developing a bank of search terms and iteratively refining these terms. Although the terms for the domain of the patient are relatively consistent, those terms relevant to the domain of benefits and risks of medicines are variable. Most search terms in the domain were extracted as free text from the screened articles. The MeSH terms of Benefit Risk Assessment and Risk Assessment were neither precise nor sensitive in retrieving articles pertinent to patient-based BRA of medicines. Specific entries corresponding to patient-based risk assessment or patient-based benefit-risk assessment were not found below these terms in the MeSH hierarchy. It seems that the concept of patient-based BRA has not yet been identified under the larger concept of risk assessment or BRA in general.

Despite the relative consistency in the use of keywords and MeSH terms in the domain of patients and their preferences, we did not incorporate any of these terms in the search syntax of our validation case of MS—with and without the proposed content search strategy. The main reason was to detect the largest possible number of publications that address patient-based BRA of drug therapy in MS. For example, the use of certain terms (e.g, Patient Preference [MeSH] OR Patient Preference / psychology [MeSH Subheading] OR Patient Preference / statistics & numerical data [MeSH Subheading] OR Decision Making [MeSH] OR Choice Behavior [MeSH] OR Health Knowledge, Attitudes, Practice [MeSH]) in the syntax for MS would have precluded retrieving articles where the keywords used to describe patient preference were respondents' attitude, patient engagement, or opinion of individuals. Screening of the many publications related to MS and its treatment in the last ten years allowed us to detect more precisely the true and—more importantly—the false negatives of the proposed content search strategy. The benchmarking of the pool of articles included in our validation versus those included in two published systematic reviews on patient-based BRA of medicines in MS permitted an optimal calculation of the performance parameters. Testing our content search strategy against a completed systematic review permitted us to calculate the relative performance of our generic search strategy versus the sophisticated and thorough research approach adopted in systematic reviews. It also allowed us to test it in a different case condition.

The success of an empirically derived content search strategy is estimated by the generalizability of the gold standard adopted in its validation. Despite being costly and time-consuming, hand-searching is still viewed as the method of choice [20]. The manual search based on MS syntax alone (43,901 hits) enabled generation of a reference set of seventy-five articles, which represented the gold standard in the validation. Nevertheless, this reference set was limited to one disease, and this might have introduced a bias in the calculation of the performance parameters. To counter this limitation, we performed a second validation based on extracted references from a relevant systematic review in another therapeutic area, RA. The systematic review–based quasi-gold standards have been increasingly used as an alternative approach for a gold standard in search filter development and validation [20].

A key point in developing search strategies is striking a balance between the sensitivity and specificity for the intended end users. It is hard to determine when a search strategy is completed, as there are no fixed measures or criteria for performance parameters for this purpose [20]. Moreover, increasingly extensive strategies may be more prone to errors. We proposed a high-performing strategy and a more extended one with higher sensitivity but where more articles would need to be read. The different performance levels of those two strategies will cater to different end users and their particular information needs.

### Strengths and limitations

This is the first study, to our knowledge, to develop a content search strategy that contains generic keywords and MeSH terms and subheadings that retrieve published data on patient-based BRA of medicines with high sensitivity, specificity, and accuracy. It can be used to identify the evidence on patient preferences and perspectives on their medicines in any therapeutic area when combined (using the Boolean operator AND) with search entries pertinent to a given disease or condition. This research-based search strategy can replace subjectively derived and unvalidated strategies currently used in the field of patient-based BRA, which rely more on expert opinions and are regarded as methodologically weak in standard biomedical evidence hierarchies [48, 49].

The main study limitation is that the search strategy was designed and validated using only one database, PubMed. All search strategies must be developed for a specific database [18], due to disparities in metadata sets and discrepancies in the use of controlled vocabularies, search syntax, truncation, and proximity operators [48]. This content search strategy could be readapted, and its performance reexamined in other large bibliographic databases (e.g., Embase).

### Implications for practice

This search strategy for identifying patient-based BRA of medicines can be used with confidence by not only information specialists but also clinicians or regulators with limited bibliographic search skills in a wide range of clinical areas where there is a need or wish to integrate patients' perspectives in the assessment of medicines. Its metric-based performance can inform prospective users of the retrieval rate they can expect in their search. An extended search strategy is also proposed to be used for a more exhaustive search. The methods used to develop and validate the search filter can also be replicated in other complex search domains.

## Data Availability

There is no additional data related to this manuscript. Endnote libraries can be shared upon request.

## SUPPLEMENTAL FILES

**Appendix 1:** Search terms forming the hedge

**Appendix 2:** Articles screened for building and refining a search term bank with corresponding keywords and MeSH terms

**Appendix 3:** Extended content search strategy and testing its relative performance by replicating a published systematic review

**Appendix 4:** Articles included in two systematic reviews on patient preferences for attributes of disease-modifying drugs in multiple sclerosis

**Appendix 5:** Testing the relative performance of the content search strategy by replicating a published systematic review

**Appendix 6:** Reproducible search strategies on patient-based benefit-risk assessment of medicines in PubMed
