# Supplementary material for: Patient-based benefit-risk assessment of medicines: development, refinement, and validation of a content search strategy to retrieve relevant studies
Source: J Med Libr Assoc. 2022 Apr 1;110(2):185–204. doi: 10.5195/jmla.2022.1306 (PMC9014953; doi:10.5195/jmla.2022.1306)
Supplement: Supplementary file 1 — Appendix 1: Search terms forming the hedge [file jmla-110-2-185-s01.pdf]

Appendix 1: Search terms forming the hedge

Patient*

Inpatient*

Outpatient

Client*

Survivor*.

User*

Consumer*

Person*

People*

Individual*

Adult*

Caregiver*

Carer*

Guardian*

Focus groups

Self-Help Groups

Support group

Community Networks

View*

Experience*

Perspective*

Perception*

Misperception

Preference*

Social perception*

Narrative*

Discourse*

Satisfaction

Perceived

Concern*

Issue*

Attitude*

Emotion*

Fear*.

Disappoint*.

Afraid

Distress

Nervous

Anxi*

Frustat*

Negativism

Unmet need*

Needs assessment [MeSH]

Stress, psychological [MeSH]

Adaptation, psychological [MeSH]

Cope

Coping

Worr*

Quality of life

Qol

Quality of recovery

Patient advocacy

Consumer advocacy

Right to choose

Choice OR Informed choice

Empower

Decision making [MeSH]

Decision making, shared [MeSH Terms]

Decision Support Techniques [Mesh]

Communication

Access to information

Information services

Self-help groups

Professional-Patient Relations [MeSH]

Patient preference*

Patient Expert

Patient Preference [MeSH]

Patient Participation [MeSH]

Patient Satisfaction [MeSH]

Consumer Satisfaction

Consumer Participation

User satisfaction

User participation

Patient desires [tiab]

Patient view*[tiab] OR patient's view*[tiab]

Patient expression*[tiab] OR patient's expression*[tiab]

Patient attitude*[tiab] OR patient's attitude*[tiab]

Patient involvement*[tiab] OR patient's involvement*[tiab]

Patient decision*[tiab] OR patient's decision*[tiab]

Patient activation[tiab] OR patient's activation[tiab] OR patients activation[tiab]

Patient empowerment [tiab]

Patient-focused [tiab] OR patient-centred [tiab] OR patient-centered [tiab]

Cooperative behavior

Self-efficacy

Self-concept

Patient Education [MeSH]

Patient-Centered Care [Mesh]

Patient Acceptance of Health Care [MeSH]

Attitude to Health [MeSH]

Risk Assessment [Mesh]

Benefit Risk Assessment [Mesh]

Risk Reduction Behavior [Mesh]
