# Supplementary material for: Patient-based benefit-risk assessment of medicines: development, refinement, and validation of a content search strategy to retrieve relevant studies
Source: J Med Libr Assoc. 2022 Apr 1;110(2):185–204. doi: 10.5195/jmla.2022.1306 (PMC9014953; doi:10.5195/jmla.2022.1306)
Supplement: Supplementary file 2 — Appendix 2: Articles screened for building and refining a search term bank with corresponding keywords and MeSH terms [file jmla-110-2-185-s02.pdf]

Appendix 2: Articles screened for building and refining a search term bank with corresponding keywords and MeSH terms

Reference	Therapeutic area	Keywords		MeSH terms	
		Patient-focused	Benefit-risk assessment of medicine	Patient-focused	Benefit-risk assessment of medicine
Welzel et al. [1]	Chronic hepatitis C	- Patient preferences	- Treatment<br>- Attributes<br>- Tolerability	- Patient Preference / psychology<br>- Patient Preference / statistics & numerical data*	
Mühlbacher et al. [2]	Hepatitis C	- Patient preferences<br>- Stated preferences	- Probability of occurrence<br>- Severity<br>- Treatment-induced side effects<br>- Attribute	- Choice Behavior*<br>- Patient Preference*	
Brett Hauber et al. [3]	Hepatitis C	- Patient preferences	- Treatment attributes<br>- The most important attribute<br>- Relative importance	- Choice Behavior<br>- Patient Preference/ psychology*	- Treatment Outcome
Simoni et al. [4]	HIV		- Side effects<br>- Dosing<br>- Efficacy<br>- Acceptable regimen	- Patient Acceptance of Health care/psycholog y*<br>- Patient preference*	
Van Houtven et al. [5]	Crohn's disease		- Benefit-risk preference<br>- Risk tolerance<br>- Benefit-risk analysis<br>- Trade-offs	- Choice Behavior*	- Risk Assessment/ methods*
Johnson et al. [6]	Crohn's disease	- Treatment preferences	- Tradeoff<br>- Maximum acceptable risk<br>- Serious adverse events<br>- Treatment attributes	- Patient Acceptance of Health Care	- Risk assessment
Hodgkins et al. [7]	Ulcerative colitis	- Patient preferences	- Attributes<br>- Effectiveness	- Choice Behavior*<br>- Patient Preference*	
Vasconcelos et al. [8]	Psoriasis	- Patient preferences	- Most important attribute<br>- Attributes	- Patient Preference*	

Schaarschmidt et al. [9]	Psoriasis	- Patient preference	- Treatment-related adverse effects<br>- Probability of benefit<br>- Attributes<br>- Relative importance	- Patient Preference*	- Outcome Assessment, Health Care / methods*
Rigopoulos et al. [10]	Psoriasis	- Treatment preferences	- Risk of SAEs<br>- Safety	- Patient Preference*	- Drug Administration Schedule
Schaarschmidt et al. [11]	Psoriasis	- Treatment preferences	- Attributes<br>- Relative importance	- Patient Preference*	- Treatment Outcome
Kromer et al. [12]	Psoriasis	- Patients' preferences<br>- Treatment preference	- Preferences for outcome<br>- Attribute	- Patient Preference / psychology*<br>- Patient Preference / statistics & numerical data*	- Treatment Outcome
Felix et al. [13]	Psoriasis	- Patients perception<br>- Willingness	- Medication side effects<br>- Quite important<br>- Extremely important	- Patient Preference / psychology	-
Rothery et al. [14]	Psoriatic arthritis	- Patient preferences	- Treatment-related benefits and risk<br>- Most important benefit attribute	- Patient Preference*<br>- Choice Behavior	- Risk<br>- Treatment Outcome
Husni et al. [15]	Rheumatoid arthritis	- Patient preference	- Trade-offs<br>- Benefit-risk trade-offs<br>- Efficacy<br>- Adverse events<br>- Attributes<br>- Benefit-risk<br>- Treatment options	- Choice Behavior<br>- Patient Preference*	- Risk Assessment<br>- Treatment Outcome
Mohamed et al. [16]	Immunoglobin treatment	- Patient preferences	- Administration<br>- Attributes<br>- Treatment profiles	- Choice Behavior<br>- Patient Preference / statistics & numerical data*	
Poulos et al. [17]	Multiple sclerosis	- Stated preference	- Treatment attributes<br>- Minimum acceptable efficacy	- Patient Preference / psychology*<br>- Choice Behavior	
Arroyo et al. [18]	Multiple sclerosis	- Patient preferences	- Most acceptable<br>- Least acceptable<br>- Medication attributes<br>- Side effect risk<br>- Route of administration<br>- Frequency of administration	- Decision Making*<br>- Patient Preference*	

			- Risk-benefit spectrum		
Poulos et al. [19]	Multiple sclerosis	- Preferences	- Tradeoffs<br>- Attribute<br>- Treatment efficacy	- Choice Behavior<br>- Patient Preference / psychology<br>- Patient Preference / statistics & numerical data*	
Wüllner et al. [20]	Parkinson’s disease	- Patient preference<br>- Patient's perspective	- Treatment regimen		- Treatment Outcome
Ettinger et al. [21]	Epilepsy	- Patient preferences	- Adverse effects<br>- Treatment attributes	- Choice Behavior*<br>- Patient Preference/psychology*	
Hauber et al. [22]	Secondary hyperparathyroidism	- Patient preferences	- Attributes<br>- Treatment attributes<br>- Efficacy<br>- Safety<br>- Tolerability<br>- Mode of administration<br>- Relative importance	- Patient Preference*	
Flood et al. [23]	Diabetes	- Patient preferences	- Treatment attributes<br>- Efficacy<br>- Regimen<br>- Risk of common side effects<br>- Rare but serious adverse events	- Patient Preference*	
Gelhorn et al. [24]	Diabetes	- Patients preference	- Attributes<br>- Side effects<br>- Efficacy<br>- Mode of administration<br>- The most important attribute	- Choice Behavior*<br>- Decision Making<br>- Patient Preference*	
Fifer et al. [25]	Diabetes	- Patient preference	- Trade-offs<br>- Attributes	- Choice Behavior<br>- Patient Preference / statistics & numerical data	
Boye et al. [26]	Diabetes	- Patients’ perspective	- Utility<br>- Attribute	-	- Drug Administration Routes<br>- Drug Administration Schedule
Janssen et al. [27]	Diabetes	- Patient preferences	-	- Choice Behavior*<br>- Decision Making<br>- Patient Preference*	

Lane et al. [28]	Atrial fibrillation	- Patient preference	- Efficacy<br>- Safety<br>- Dosing frequency<br>- Attribute	- Patient Preference*	- Risk assessment<br>- Treatment outcome
Edwards et al. [29]	Atrial fibrillation	- Patient preference	- Medication-related factors<br>- Medication preference<br>- Most preferred treatment options,<br>- Least preferred treatment options	- Patient Preference / psychology*	
Weernink et al. [30]	Atrial fibrillation	- Perspective of patients<br>- Patient preferences	- Attributes<br>- Effectiveness<br>- Safety<br>- Side effects	- Patient Preference	
Mühlbacher et al. [31]	Acute coronary syndrome		- Treatment attributes	- Choice Behavior*<br>- Decision Making*<br>- Patient Preference	- Risk
Fletcher et al. [32]	Hypertension	Patient preferences	- Attribute<br>- Risk reduction<br>- Relative importance	- Choice Behavior<br>- Decision Making<br>- Patient Preference*	- Risk Reduction Behavior*
Wright et al. [33]	Anticoagulation	- Preference	- Effectiveness<br>- Value<br>- Benefit	- Patient Preference / statistics & numerical data*	-
Najafzadeh et al. [34]	Anticoagulation	- Choice behavior	- Benefit-risk assessment<br>- Patient centered outcome<br>- Utility	- Choice Behavior*<br>- Patient Preference / psychology*	- Risk Assessment
Lutsey et al., [35]	Venous thromboembolism	- Patient concerns<br>- Patient preferences	- Characteristics	- Patient Preference*	
Noble et al. [36]	Cancer-associated thrombosis	- Perception	- Acceptable trade-off<br>- Attributes<br>- Efficacy<br>- Safety<br>- Route of administration	- Patient Preference*	
Postmus et al. [37]	Multiple myeloma	- Patient preferences	- Severe toxicity<br>- Life-threatening toxicity<br>- Acceptability<br>- Attributes<br>- Benefit-risk assessment	- Patient Preference / psychology,<br>- Patient Preference / statistics & numerical data*	- Risk Assessment / methods*
Kaehler et al. [38]	Melanoma		- Trade-offs<br>- Side effects<br>- Therapeutic benefit	- Patient Preference* / psychology	- Drug-Related Side Effects and Adverse

			- Patient utilities<br>- Health states	- Patient Preference* / statistics & numerical data	Reactions* / psychology,<br>- Outcome and Process Assessment, Health Care
Mühlbacher et al. [39]	Lung cancer	- Patients' preference	- Patient-relevant treatment attributes<br>- Efficacy<br>- Side effects<br>- Mode of administration	- Choice Behavior<br>- Patient preference*	
Islam et al. [40]	Lung cancer	- Treatment choices	- Regimen<br>- Side effects	- Patient preference*	
Marshall et al. [41]	Breast cancer	- Patient preferences	- Benefit-risk trade-offs<br>- Attributes	- Patient Preference,<br>- Choice Behavior*<br>- Decision Making*	- Risk Assessment
Kuchuk et al. [42]	Breast cancer	- Perceptions	- Relative importance<br>- Gamble<br>- Utilities	- Patient Preference*	- Drug-Related Side Effects and Adverse Reactions / psychology*
Omori et al. [43]	Breast cancer	- Patients' preferences	- Attributes	- Patient Preference*	
Lee et al. [44]	Ovarian cancer	- Patient preference<br>- Treatment preference	- Trade-off<br>- Effectiveness<br>- Safety	- Choice Behavior*<br>- Patient Preference*	
Havrilesky et al. [45]	Ovarian cancer	- Patient preferences	- Benefit<br>- Risk	- Patient Preference / psychology*	
Sun et al. [46]	Cervical cancer	- Patient preferences	- Treatment-related adverse effects	- Patient Preference*	- Combined Modality Therapy/adverse effects*
Hobden et al. [47]	Depression in cancer	- Concern	- Most preferred options<br>- Least preferred options	- Patient Preference / psychology*	
Rosenblat et al. [48]	Depression		- Treatment effectiveness<br>- Tolerability<br>- Patient-centred outcomes<br>- Adverse effects<br>- Patient reported outcomes (PROs)	-	- Patient Reported Outcome Measures*<br>- Treatment Outcome
Eiring et al. [49]	Bipolar disorder	- Patients' preferences	- Relative importance	- Patient Preference / psychology*<br>- Patient Preference / statistics & numerical data	- Treatment Outcome*

Jørgensen et al. [50]	Bipolar disorder and schizophrenia	- Patient preferences	- Preferences for medication attributes	- Patient Preference*	- Drug Administration Routes*
Achtyes et al. [51]	Schizophrenia	- Patient concerns	- Efficacy<br>- Side effects<br>- Most important factors	- Patient preference*	- Drug-related side effects and adverse reactions/psychology
Levitan et al. [52]	Schizophrenia	- Patients' preferences	- Benefits and risks<br>- The most important treatment benefit<br>- The most important adverse event	- Choice Behavior*<br>- Patient Preference*	
Cheung et al. [53]	Insomnia	- Patient preferences	- Treatment attributes<br>- Long-term benefits<br>- Duration of treatment course	- Choice Behavior*<br>- Decision Making*<br>- Patient Preference / psychology*	
Gelhorn et al. [54]	Asthma	- Patient preference	- Attribute<br>- Administration<br>- Dosing		
Svedsater et al. [55]	Asthma or COPD	- Treatment preferences	- Burden<br>- Treatment attributes	- Patient Preference*<br>- Choice Behavior*	
Mohamed et al. [56]	Cystic fibrosis		- Attributes<br>- Frequency of administration<br>- Administration time	- Patient Preference*	
Billington et al. [57]	Osteoporosis	- Experience<br>- Patient preference	- Value		
Hiligsmann et al. [58]	Osteoporosis	- Patients preferences	- Medication attributes<br>- Drug treatment	- Attitude to Health, Patient Preference*	- Risk Assessment
Mattsson et al. [59]	Vaginal atrophy	- Preference	-	- Attitude to health<br>- Patient preference*	
Anstey Watkins et al. [60]	Bacterial vaginosis	- Concern<br>- Treatment preference	- Acceptability<br>- Burden<br>- Effectiveness<br>- Tolerability	- Patient Preference*<br>- Patient Acceptance of Health Care*	
van den Wijngaard et al. [61]	In vitro fertilization	- Patient preference	- Attributes<br>- Trade-off	- Choice Behavior<br>- Patient Preference*	
Agyei-Baffour et al. [62]	Contraception		- Attribute<br>- Utility<br>- Trade-offs	- Choice Behavior*<br>- Patient Preference*	
Poulos et al. [63]	Endometriosis pain	- Patient preferences	- Benefit-risk preference<br>- Risk tolerance	- Patient Preference / psychology*)	

			- Maximum acceptable risk		
Kløjgaard et al. [64]	Low back pain	- Patient preferences<br>- Stated preferences	- Treatment attribute<br>- Disutility	- Choice Behavior*<br>- Patient Preference*	- Treatment Outcome
Mühlbacher et al. [65]	Chronic pain	- Patient-oriented treatment	- Attributes	- Choice Behavior<br>- Patient Preference*	-
Iihara et al. [66]	Chronic conditions	- Patient preference	- Adverse reaction	- Patient Preference*	- Drug-Related Side Effects and Adverse Reactions* / prevention & control,<br>- Drug therapy
Morel et al.[67]	Rare diseases	- Patients<br>- Preferences	- Benefit-risk<br>- Patient-centered outcomes<br>- Patient-reported outcomes<br>- Risk tolerance<br>- Trade-offs<br>- Values	- Patient Preference / psychology*<br>- Choice Behavior	- Risk Assessment
Kopasker et al. [68]	Actinic keratoses	- Patient perspective<br>- Patient willingness	- Benefit<br>- Burden<br>- Treatment regimen<br>- Acceptability	- Patient Preference / psychology*<br>- Choice Behavior*	- Treatment Outcome
Christiansen et al. [69]	Hereditary angioedema	- Perceptions	- Medication side effects<br>- Burden	- Perception*	- Drug-Related Side Effects and Adverse Reactions / psychology<br>- Treatment Outcome
Glenngård et al. [70]	Attention deficit hyperactivity disorder	- Patient preferences	- Attribute<br>- Effectiveness<br>- Side effects<br>- Dosing	- Choice Behavior<br>- Patient Preference* / economics	

References

1. Welzel TM, Yang M, Sajeev G, Chen YJ, Pinsky B, Bao Y, Wu EQ, Dieterich D. Assessing patient preferences for treatment decisions for new direct acting antiviral (daa) therapies for chronic hepatitis c virus infections. Adv Ther. 2019;36(9):2475–86.
2. Mühlbacher A, Bethge S. First and foremost battle the virus: eliciting patient preferences in antiviral therapy for hepatitis c using a discrete choice experiment. Value Health. 2016;19(6):776–87.
3. Brett Hauber A, Mohamed AF, Beam C, Medjedovic J, Mauskopf J. Patient preferences and assessment of likely adherence to hepatitis C virus treatment. J Viral Hepat. 2011;18(9):619–27.
4. Simoni JM, Beima-Sofie K, Mohamed ZH, Christodoulou J, Tapia K, Graham SM, Ho R, Collier AC. Long-acting injectable antiretroviral treatment acceptability and preferences: a qualitative study among US providers, adults living with HIV, and parents of youth living with HIV. AIDS Patient Care STDS. 2019;33(3):104–11.
5. Van Houtven G, Johnson FR, Kilambi V, Hauber AB. Eliciting benefit-risk preferences and probability-weighted utility using choice-format conjoint analysis. Med Decis Making. 2011;31(3):469–80.
6. Johnson FR, Hauber B, Özdemir S, Siegel CA, Hass S, Sands BE. Are gastroenterologists less tolerant of treatment risks than patients? Benefit-risk preferences in Crohn's disease management. J Manag Care Pharm. 2010;16(8):616–28.
7. Hodgkins P, Swinburn P, Solomon D, Yen L, Dewilde S, Lloyd A. Patient preferences for first-line oral treatment for mild-to-moderate ulcerative colitis: a discrete-choice experiment. Patient. 2012;5(1):33–44.
8. Vasconcelos V, Teixeira A, Almeida V, Teixeira M, Ramos S, Torres T, Sousa Lobo JM, Almeida IF. Patient preferences for attributes of topical anti-psoriatic medicines. J Dermatolog Treat. 2019;30(7):659–63.
9. Schaarschmidt ML, Schmieder A, Umar N, Terris D, Goebeler M, Goerdt S, Peitsch WK. Patient preferences for psoriasis treatments: process characteristics can outweigh outcome attributes. Arch Dermatol. 2011;147(11):1285–94.
10. Rigopoulos D, Ioannides D, Chaidemenos G, Kallidis P, Voultsidou A, Matekovits A, Soura E. Patient preference study for different characteristics of systemic psoriasis treatments (Protimisis). Dermatol Ther. 2018;31(3):e12592.
11. Schaarschmidt ML, Umar N, Schmieder A, Terris DD, Goebeler M, Goerdt S, Peitsch WK.. Patient preferences for psoriasis treatments: impact of treatment experience. J Eur Acad Dermatol Venereol. 2013;27(2):187–98.
12. Kromer C, Peitsch WK, Herr R, Schmieder A, Sonntag D, Schaarschmidt ML. Treatment preferences for biologicals in psoriasis: experienced patients appreciate sustainability. J Dtsch Dermatol Ges. 2017;15(2):189–200.
13. Felix K, Unrue E, Inyang M, Cardwell LA, Oussedik E, Richardson I, Feldman SR. Patients preferences for different corticosteroid vehicles are highly variable. J Dermatolog Treat. 2020;31(2):147–51.
14. Rothery C, Bojke L, Richardson G, Bojke C, Moverley A, Coates L, Thorp L, Waxman R, Helliwell P. A discrete choice experiment to explore patients' willingness to risk disease relapse from treatment withdrawal in psoriatic arthritis. Clin Rheumatol. 2016;35(12):2967–74.
15. Husni ME, Betts KA, Griffith J, Song Y, Ganguli A. Benefit-risk trade-offs for treatment decisions in moderate-to-severe rheumatoid arthritis: focus on the patient perspective. Rheumatol Int. 2017;37(9):1423–34.
16. Mohamed AF, Kilambi V, Luo MP, Iyer RG, Li-McLeod JM. Patient and parent preferences for immunoglobulin treatments: a conjoint analysis. J Med Econ. 2012;15(6):1183–91.
17. Poulos C, Kinter E, van Beek J, Christensen K, Posner J. Preferences of patients with multiple sclerosis for attributes of injectable multiple sclerosis treatments in the United Kingdom and France. Int J Technol Assess Health Care. 2018;34(4):425–33.

18. Arroyo R, Sempere AP, Ruiz-Beato E, Prefasi D, Carreño A, Roset M, Maurino J. Conjoint analysis to understand preferences of patients with multiple sclerosis for disease-modifying therapy attributes in Spain: a cross-sectional observational study. BMJ Open. 2017;7(3):e014433.
19. Poulos C, Kinter E, Yang JC, Bridges JF, Posner J, Reder AT. Patient preferences for injectable treatments for multiple sclerosis in the United States: a discrete-choice experiment. Patient. 2016;9(2):171–80.
20. Wüllner U, Fuchs G, Reketat N, Randerath O, Kassubek J. Requirements for Parkinson's disease pharmacotherapy from the patients' perspective: a questionnaire-based survey. Curr Med Res Opin. 2012;28(7):1239–46.
21. Ettinger AB, Carter JA, Rajagopalan K. Patient versus neurologist preferences: a discrete choice experiment for antiepileptic drug therapies. Epilepsy Behav. 2018;80:247–53.
22. Hauber B, Caloyeras J, Posner J, Brommage D, Belozeroff V, Cooper K. Hemodialysis patients' preferences for the management of secondary hyperparathyroidism. BMC Nephrol. 2017;18(1):254.
23. Flood EM, Bell KF, de la Cruz MC, Ginchereau-Sowell FM. Patient preferences for diabetes treatment attributes and drug classes. Curr Med Res Opin. 2017;33(2):261–8.
24. Gelhorn HL, Stringer SM, Brooks A, Thompson C, Monz BU, Boye KS, Hach T, Lund SS, Palencia R. Preferences for medication attributes among patients with type 2 diabetes mellitus in the UK. Diabetes Obes Metab. 2013;15(9):802–9.
25. Fifer S, Rose J, Hamrosi KK, Swain D. Valuing injection frequency and other attributes of type 2 diabetes treatments in Australia: a discrete choice experiment. BMC Health Serv Res. 2018;18(1):675.
26. Boye KS, Matza LS, Walter KN, Van Brunt K, Palsgrove AC, Tynan A. Utilities and disutilities for attributes of injectable treatments for type 2 diabetes. Eur J Health Econ. 2011;12(3):219–30.
27. Janssen EM, Hauber AB, Bridges JFP. Conducting a discrete-choice experiment study following recommendations for good research practices: an application for eliciting patient preferences for diabetes treatments. Value Health. 2018;21(1):59–68.
28. Lane DA, Meyerhoff J, Rohner U, Lip GYH. Atrial fibrillation patient preferences for oral anticoagulation and stroke knowledge: results of a conjoint analysis. Clin Cardiol. 2018;41(6):855–61.
29. Edwards NT, Greanya ED, Kuo IF, Loewen PS, Culley CL. Patient preferences regarding atrial fibrillation stroke prophylaxis in patients at potential risk of atrial fibrillation. Int J Clin Pharm. 2017;39(2):468–72.
30. Weernink MGM, Vaanholt MCW, Groothuis-Oudshoorn CGM, von Birgelen C, MJ IJ, van Til JA. Patients' priorities for oral anticoagulation therapy in non-valvular atrial fibrillation: a multi-criteria decision analysis. Am J Cardiovasc Drugs. 2018;18(6):493–502.
31. Mühlbacher AC, Bethge S. Reduce mortality risk above all else: a discrete-choice experiment in acute coronary syndrome patients. Pharmacoeconomics. 2015;33(1):71–81.
32. Fletcher B, Hinton L, McManus R, Rivero-Arias O. Patient preferences for management of high blood pressure in the UK: a discrete choice experiment. Br J Gen Pract. 2019;69(686):e629–e637.
33. Wright JN, Vazquez SR, Kim K, Jones AE, Witt DM. Assessing patient preferences for switching from warfarin to direct oral anticoagulants. J Thromb Thrombolysis. 2019;48(4):596–602.
34. Najafzadeh M, Gagne JJ, Choudhry NK, Polinski JM, Avorn J, Schneeweiss SS. Patients' preferences in anticoagulant therapy: discrete choice experiment. Circ Cardiovasc Qual Outcomes. 2014;7(6):912–9.
35. Lutsey PL, Horvath KJ, Fullam L, Moll S, Rooney MR, Cushman M, Zakai NA. Anticoagulant preferences and concerns among venous thromboembolism patients. Thromb Haemost. 2018;118(3):553–61.
36. Noble S, Matzdorff A, Maraveyas A, Holm MV, Pisa G. Assessing patients' anticoagulation preferences for the treatment of cancer-associated thrombosis using conjoint methodology. Haematologica. 2015;100(11):1486–92.

37. Postmus D, Richard S, Bere N, van Valkenhoef G, Galinsky J, Low E, Moulon I, Mavris M, Salmonsson T, Flores B, Hillege H, Pignatti F. Individual trade-offs between possible benefits and risks of cancer treatments: results from a stated preference study with patients with multiple myeloma. Oncologist. 2018;23(1):44–51.
38. Kaehler KC, Blome C, Forschner A, Gutzmer R, Haalck T, Heinzerling L, Kornek T, Livingstone E, Loquai C, Maul LV, Lang BM, Schadendorf D, Stade B, Terheyden P, Utikal J, Wagner T, Hauschild A, Garbe C, Augustin M. Preferences of German melanoma patients for interferon (IFN) α-2b toxicities (the DeCOG "GERMELATOX survey") versus melanoma recurrence to quantify patients' relative values for adjuvant therapy. Medicine (Baltimore). 2016;95(46):e5375.
39. Mühlbacher AC, Bethge S. Patients' preferences: a discrete-choice experiment for treatment of non-small-cell lung cancer. Eur J Health Econ. 2015;16(6):657–70.
40. Islam KM, Anggondowati T, Deviany PE, Ryan JE, Fetrick A, Bagenda D, Copur MS, Tolentino A, Vaziri I, McKean HA, Dunder S, Gray JE, Huang C, Ganti AK. Patient preferences of chemotherapy treatment options and tolerance of chemotherapy side effects in advanced stage lung cancer. BMC Cancer. 2019;19(1):835.
41. Marshall DA, Deal K, Bombard Y, Leighl N, MacDonald KV, Trudeau M. How do women trade-off benefits and risks in chemotherapy treatment decisions based on gene expression profiling for early-stage breast cancer? A discrete choice experiment. BMJ Open. 2016;6(6):e010981.
42. Kuchuk I, Bouganim N, Beusterien K, Grinspan J, Vandermeer L, Gertler S, Dent SF, Song X, Segal R, Mazzarello S, Crawley F, Dranitsaris G, Clemons M. Preference weights for chemotherapy side effects from the perspective of women with breast cancer. Breast Cancer Res Treat. 2013;142(1):101–7.
43. Omori Y, Enatsu S, Cai Z, Ishiguro H. Patients' preferences for postmenopausal hormone receptor-positive, human epidermal growth factor receptor 2-negative advanced breast cancer treatments in Japan. Breast Cancer. 2019;26(5):652–62.
44. Lee JY, Kim K, Lee YS, Kim HY, Nam EJ, Kim S, Kim SW, Kim JW, Kim YT. Treatment preferences of advanced ovarian cancer patients for adding bevacizumab to first-line therapy. Gynecol Oncol. 2016;143(3):622–7.
45. Havrilesky LJ, Lim S, Ehrisman JA, Lorenzo A, Alvarez Secord A, Yang JC, Johnson FR, Gonzalez JM, Reed SD. Patient preferences for maintenance PARP inhibitor therapy in ovarian cancer treatment. Gynecol Oncol. 2020;156(3):561–7.
46. Sun C, Brown AJ, Jhingran A, Frumovitz M, Ramondetta L, Bodurka DC. Patient preferences for side effects associated with cervical cancer treatment. Int J Gynecol Cancer. 2014;24(6):1077–84.
47. Hobden B, Turon H, Bryant J, Wall L, Brown S, Sanson-Fisher R. Oncology patient preferences for depression care: a discrete choice experiment. Psychooncology. 2019;28(4):807–14.
48. Rosenblat JD, Simon GE, Sachs GS, Deetz I, Doederlein A, DePeralta D, Dean MM, McIntyre RS. Treatment effectiveness and tolerability outcomes that are most important to individuals with bipolar and unipolar depression. J Affect Disord. 2019;243:116–20.
49. Eiring Ø, Nylenna M, Nytrøen K. Patient-important outcomes in the long-term treatment of bipolar disorder: a mixed-methods approach investigating relative preferences and a proposed taxonomy. Patient. 2016;9(2):91–102.
50. Jørgensen TR, Emborg C, Dahlen K, Bøgelund M, Carlborg A. Patient preferences for medicine administration for acute agitation: results from an internet-based survey of patients diagnosed with bipolar disorder or schizophrenia in two Nordic countries. Psychol Health Med. 2018;23(1):30–8.
51. Achtyes E, Simmons A, Skabeev A, Levy N, Jiang Y, Marcy P, Weiden PJ. Patient preferences concerning the efficacy and side-effect profile of schizophrenia medications: a survey of patients living with schizophrenia. BMC Psychiatry. 2018;18(1):292.
52. Levitan B, Markowitz M, Mohamed AF, Johnson FR, Alphs L, Citrome L, Bridges JF. Patients' preferences related to benefits, risks, and formulations of schizophrenia treatment. Psychiatr Serv. 2015;66(7):719–26.

53. Cheung JMY, Bartlett DJ, Armour CL, Saini B, Laba TL. Patient preferences for managing insomnia: a discrete choice experiment. Patient. 2018;11(5):503–14.
54. Gelhorn HL, Balantac Z, Ambrose CS, Chung YN, Stone B. Patient and physician preferences for attributes of biologic medications for severe asthma. Patient Prefer Adherence. 2019;13:1253–68.
55. Svedsater H, Leather D, Robinson T, Doll H, Nafees B, Bradshaw L. Evaluation and quantification of treatment preferences for patients with asthma or COPD using discrete choice experiment surveys. Respir Med. 2017;132:76–83.
56. Mohamed AF, Johnson FR, Balp MM, Calado F. Preferences and stated adherence for antibiotic treatment of cystic fibrosis pseudomonas infections. Patient. 2016;9(1):59–67.
57. Billington EO, Feasel AL, Kline GA. At odds about the odds: women's choices to accept osteoporosis medications do not closely agree with physician-set treatment thresholds. J Gen Intern Med. 2020;35(1):276–82.
58. Hiligsmann M, Dellaert BG, Dirksen CD, Watson V, Bours S, Goemaere S, Reginster JY, Roux C, McGowan B, Silke C, Whelan B, Diez-Perez A, Torres E, Papadakis G, Rizzoli R, Cooper C, Pearson G, Boonen A. Patients' preferences for anti-osteoporosis drug treatment: a cross-European discrete choice experiment. Rheumatology (Oxford). 2017;56(7):1167–76.
59. Mattsson L, Ericsson Å, Bøgelund M, Maamari R. Women's preferences toward attributes of local estrogen therapy for the treatment of vaginal atrophy. Maturitas. 2013;74(3):259–63.
60. Anstey Watkins J, Ross JDC, Thandi S, Brittain C, Kai J, Griffiths F. Acceptability of and treatment preferences for recurrent bacterial vaginosis-Topical lactic acid gel or oral metronidazole antibiotic: qualitative findings from the VITA trial. PLoS One. 2019;14(11):e0224964.
61. van den Wijngaard L, Rodijk IC, van der Veen F, Gooskens-van Erven MH, Koks CA, Verhoeve HR, Mol BWJ, van Wely M, Mochtar MH. Patient preference for a long-acting recombinant FSH product in ovarian hyperstimulation in IVF: a discrete choice experiment. Hum Reprod. 2015;30(2):331–7.
62. Agyei-Baffour P, Boahemaa MY, Addy EA. Contraceptive preferences and use among auto artisanal workers in the informal sector of Kumasi, Ghana: a discrete choice experiment. Reprod Health. 2015;12:32.
63. Poulos C, Soliman AM, Renz CL, Posner J, Agarwal SK. Patient preferences for endometriosis pain treatments in the United States. Value Health. 2019;22(6):728–38.
64. Kløjgaard ME, Manniche C, Pedersen LB, Bech M, Søgaard R. Patient preferences for treatment of low back pain-a discrete choice experiment. Value Health. 2014;17(4):390–6.
65. Mühlbacher AC, Junker U, Juhnke C, Stemmler E, Kohlmann T, Leverkus F, Nübling M. Chronic pain patients' treatment preferences: a discrete-choice experiment. Eur J Health Econ. 2015;16(6):613–28.
66. Iihara N, Ohara E, Nishio T, Muguruma H, Matsuoka E, Houchi H, Kirino Y. Patient preference for aggressive medication therapies with potentially stronger adverse drug reactions revealed using a scenario-based survey. Yakugaku Zasshi. 2017;137(9):1161–7.
67. Morel T, Aymé S, Cassiman D, Simoens S, Morgan M, Vandebroek M. Quantifying benefit-risk preferences for new medicines in rare disease patients and caregivers. Orphanet J Rare Dis. 2016;11(1):70.
68. Kopasker D, Kwiatkowski A, Matin RN, Harwood CA, Ismail F, Lear JT, Thomson J, Hasan Z, Wali GN, Milligan A, Crawford L, Ahmed I, Duffy H, Proby CM, Allanson PF. Patient preferences for topical treatment of actinic keratoses: a discrete-choice experiment. Br J Dermatol. 2019;180(4):902–9.
69. Christiansen SC, Bygum A, Banerji A, Busse P, Li H, Lumry W, Davis-Lorton M, Bernstein JA, Frank MM, Castaldo A, Long JF, Riedl M, Zuraw BL. Before and after, the impact of available on-demand treatment for HAE. Allergy Asthma Proc. 2015;36(2):145–50.

70. Glenngård AH, Hjelmgren J, Thomsen PH, Tvedten T. Patient preferences and willingness-to-pay for ADHD treatment with stimulants using discrete choice experiment (DCE) in Sweden, Denmark and Norway. Nord J Psychiatry. 2013;67(5):351–9.
