# Supplementary material for: Patient-based benefit-risk assessment of medicines: development, refinement, and validation of a content search strategy to retrieve relevant studies
Source: J Med Libr Assoc. 2022 Apr 1;110(2):185–204. doi: 10.5195/jmla.2022.1306 (PMC9014953; doi:10.5195/jmla.2022.1306)
Supplement: Supplementary file 3 — Appendix 3: Extended content search strategy and testing its relative performance by replicating a published systematic review [file jmla-110-2-185-s03.pdf]

Appendix 3. Extended content search strategy and testing its relative performance by replicating a published systematic review.

Table 1: Extended content search strategy

Patient-based	Benefit-risk assessment
Patient Preference [MeSH]<br>Patient Preference / psychology [MeSH Subheading]<br>Patient Preference / statistics & numerical data [MeSH Subheading]<br>Patient Preference* [tiab]<br>Patients preference [tiab]<br>Patient perception*[tiab]<br>Perception [tiab]<br>Stated preference* [tiab]<br>Treatment preference [tiab]<br>Treatment satisfaction [tiab]<br>Willingness [tiab]<br>Willingness to pay [All fields]<br>Patient concerns [tiab]<br>Choice Behavior [MeSH]<br>Decision Making [MeSH]<br>Health Knowledge, Attitudes, Practice [MeSH]<br>Attitude to Health [MeSH]<br>Patient Acceptance of Health Care [MeSH]<br>Patient Acceptance of Health Care/psychology*[MeSH]	Attribute [tiab]<br>Benefit [tiab]<br>Benefit-risk [All fields]<br>Risk tolerance [All fields]<br>Risk awareness [All fields]<br>Risk perception [All fields]<br>Trade-off* [All fields]<br>Tradeoff* [All fields]<br>Efficacy [tiab]<br>Safety [tiab]<br>Side effect* [tiab]<br>Adverse event* [tiab]<br>Adverse reaction* [tiab]<br>Probability of occurrence [tiab]<br>Effectiveness [tiab]<br>Frequency [tiab]<br>Value [tiab]<br>Utility [tiab]<br>Disutility [tiab]<br>Accepta* [tiab]<br>Maximum acceptable risk [All fields]<br>Minimum acceptable efficacy [All fields]<br>Acceptable regimen [tiab]<br>Preferred treatment option [tiab]<br>Patient-reported outcome* [tiab]<br>Relative importance [tiab]

	Most preferred [tiab]<br>Least preferred [tiab]<br>Medication belie*[tiab]<br>Discontinuation [tiab]<br>Standard gamble[tiab]<br>Discrete choice experiment [tiab]<br>Conjoint analysis [tiab]<br>Benefit risk assessment [MeSH]<br>Risk Assessment [MeSH]<br>Risk Reduction Behavior[MeSH]<br>Drug-related side effects and adverse reactions/psychology [MeSH]<br>Risk [MeSH]<br>Treatment Outcome [MeSH]<br>Drug Administration Routes [MeSH]<br>Drug Administration Schedule [MeSH]<br>Outcome and Process Assessment, Health Care [MeSH]<br>Outcome Assessment, Health Care / methods [MeSH]

Table 2: Articles included in the systematic review on patient preferences for treatment of lung cancer [1] with corresponding keywords and MeSH terms

Reference	D*	ND*	Keywords / Free text words		MeSH terms	
			Patient-focused	Benefit-risk assessment of medicines	Patient-focused	Benefit-risk assessment of medicines
Kind et al. [2]		x	Health-state	Utility	Health Status	
Johnson et al. [3]	x			Side effects<br>Relative importance<br>Conjoint analysis	Patient Satisfaction	
Nafess et al. [4]	x			Utility	Attitude to Health	Treatment Outcome<br>Drug-Related Side Effects and Adverse Reactions / psychology
Gironés et al. [5]	x		Patient preferences<br>Patients' attitude	Treatment options<br>Benefit<br>Toxicities	Patient Preference<br>Decision Making<br>Choice Behavior / physiology	
Bridges et al. [6]	x		Patients' preferences<br>Treatment preferences	Attributes<br>Benefits<br>Risks<br>Conjoint analysis	Patient Preference	Treatment Outcome
Miller et al. [7]	x		Willingness to pay	Attribute<br>Value<br>Acceptable<br>Discrete-choice conjoint survey	Decision Making<br>Patient Acceptance of Health Care	
Mühlbacher et al. [8]	x		Patient preferences	Attributes<br>Efficacy<br>Side effects<br>Mode of administration<br>Discrete-choice experiment	Patient Preference<br>Choice Behavior	
Lehman et al. [9]	x		Patient preferences	Attribute<br>Benefit<br>Acceptable toxicity<br>Discrete choice experiment	Patient Preference	
Tong et al. [10]	x		Patient preferences	Attributes<br>Treatment modalities<br>Conjoint analysis	Patient Preference<br>Patient Participation<br>Decision Making	Risk
Fallowfield et al. [11]	x		Decision making	Benefit<br>Toxicity<br>Therapeutic aim	Decision Making	
Schmidt et al. [12]	x		Patient preferences<br>Treatment preferences	Attributes<br>Discrete choice experiment		
Bridges et al. [13]	x		Patient preferences	Attributes<br>Efficacy<br>Side effects<br>Dosing regimen	Patient Preference / psychology<br>Patient Preference / statistics & numerical data	
Sullivan et al. [14]	x		Treatment preferences	Attributes<br>Values	Patient Participation<br>Patient Preference / statistics & numerical data	
Sun et al. [15]	x		Patient preferences	Attributes<br>Risk-benefit		

			Treatment preferences<br>Willingness to pay	Discrete choice experiment		
Valenti et al. [16]	x		Patient preferences<br>Willingness	Attributes<br>Trade-off<br>Benefit<br>Adverse events<br>Conjoint analysis	Patient Preference	

*D: Detected by the extended content search strategy combined, using AND, with a search string relevant to lung neoplasm and its treatment: Lung Neoplasm [MeSH] OR Antineoplastic Agents [MeSH] OR lung cancer [tiab]

**ND: Not detected by the extended content search strategy combined, using AND, with a search string relevant to lung neoplasm and its treatment: Lung Neoplasm [MeSH] OR Antineoplastic Agents [MeSH] OR lung cancer [tiab]

Table 3: Quality assessment of included studies in the systematic review on patient preferences for treatment of lung cancer [1], based on a practical tool developed to critically assess patient preference studies across methodologies [17]

Reference	External validity	Quality of construct representation	Minimization of the risk of construct-irrelevant variance	Quality of reporting and analysis	Other aspects that strengthen or weaken the study	Overall quality
Kind et al. [2]	High	Medium	Moderate	Low	No difference	Medium
Johnson et al. [3]	High	Medium	High	High	Weaken	Medium
Nafess et al. [4]	Medium	High	High	High	Weaken	Medium
Gironés et al. [5]	Medium	Low	Low	High	No difference	Medium
Bridges et al. [6]	Medium	High	Moderate	High	Weaken	Medium
Miller et al. [7]	Medium	High	High	High	Weaken	Medium
Mühlbacher et al. [8]	High	High	High	High	Weaken	High
Lehman et al. [9]	Low	Medium	Low	High	No difference	Medium
Tong et al. [10]	Medium	Low	Low	High	No difference	Medium
Fallowfield et al. [11]	Medium	Low	Low	High	Weaken	Low
Schmidt et al. [12]	Medium	High	High	High	No difference	High
Bridges et al. [13]	Low	High	Medium	High	No difference	Medium
Sullivan et al. [14]	Medium	Medium	Low	High	No difference	Medium
Sun et al. [15]	High	High	High	High	No difference	High
Valenti et al. [16]	Medium	Low	Low	High	No difference	Medium

References:

1. Sugitani Y, Sugitani N, Ono S. Quantitative preferences for lung cancer treatment from the patients' perspective: a systematic review. Patient. 2020;13(5):521–36.
2. Kind P, Macran S. Eliciting social preference weights for Functional Assessment of Cancer Therapy-Lung health states. Pharmacoeconomics. 2005;23(11):1143–53.
3. Johnson FR, Hauber AB, Osoba D, Hsu MA, Coombs J, Copley-Merriman C. Are chemotherapy patients' HRQoL importance weights consistent with linear scoring rules? A stated-choice approach. Qual Life Res. 2006;15(2):285–98.
4. Nafees B, Stafford M, Gavriel S, Bhalla S, Watkins J. Health state utilities for non small cell lung cancer. Health Qual Life Outcomes. 2008;6:84.
5. Gironés R, Torregrosa D, Gómez-Codina J, Maestu I, Tenias JM, Rosell R. Lung cancer chemotherapy decisions in older patients: the role of patient preference and interactions with physicians. Clin Transl Oncol. 2012;14(3):183–9.
6. Bridges JF, Mohamed AF, Finnern HW, Woehl A, Hauber AB. Patients' preferences for treatment outcomes for advanced non-small cell lung cancer: a conjoint analysis. Lung Cancer. 2012;77(1):224–31.
7. Miller PJ, Balu S, Buchner D, Walker MS, Stepanski EJ, Schwartzberg LS. Willingness to pay to prevent chemotherapy induced nausea and vomiting among patients with breast, lung, or colorectal cancer. J Med Econ. 2013;16(10):1179–89.
8. Mühlbacher AC, Bethge S. Patients' preferences: a discrete-choice experiment for treatment of non-small-cell lung cancer. Eur J Health Econ. 2015;16(6):657–70.
9. Lehman M, Gorayski P, Watson S, Edeling D, Jackson J, Whitty J. Patient preferences regarding prophylactic cranial irradiation: a discrete choice experiment. Radiother Oncol. 2016;121(2):225–31.
10. Tong BC, Wallace S, Hartwig MG, D'Amico TA, Huber JC. Patient preferences in treatment choices for early-stage lung cancer. Ann Thorac Surg. 2016;102(6):1837–44.
11. Fallowfield LJ, Catt SL, May SF, Matthews L, Shilling VM, Simcock R, Westwell S, Jenkins VA. Therapeutic aims of drugs offering only progression-free survival are misunderstood by patients, and oncologists may be overly optimistic about likely benefits. Support Care Cancer. 2017;25(1):237–44.
12. Schmidt K, Damm K, Vogel A, Golpon H, Manns MP, Welte T, von der Schulenburg JMG. Therapy preferences of patients with lung and colon cancer: a discrete choice experiment. Patient Prefer Adherence. 2017;11:1647–56.
13. Bridges JF, la Cruz M, Pavilack M, Flood E, Janssen EM, Chehab N, Fernandes AW. Patient preferences for attributes of tyrosine kinase inhibitor treatments for EGFR mutation-positive non-small-cell lung cancer. Future Oncol. 2019;15(34):3895–907.
14. Sullivan DR, Eden KB, Dieckmann NF, Golden SE, Vranas KC, Nugent SM, Slatore CG. Understanding patients' values and preferences regarding early stage lung cancer treatment decision making. Lung Cancer. 2019;131:47–57.
15. Sun H, Wang H, Xu N, Li J, Shi J, Zhou N, Ni M, Hu X, Chen Y. Patient preferences for chemotherapy in the treatment of non-small cell lung cancer: a multicenter discrete choice experiment (DCE) study In China. Patient Prefer Adherence. 2019;13:1701–9.
16. Valentí V, Ramos J, Pérez C, Capdevila L, Ruiz I, Tikhomirova L, Sánchez M, Juez I, Llobera M, Sopena E, Rubió J, Salazar R. Increased survival time or better quality of life? Trade-off between benefits and adverse events in the systemic treatment of cancer. Clin Transl Oncol. 2020;22(6):935–42.
17. Eiring Ø, Landmark BF, Aas E, Salkeld G, Nylenna M, Nytrøen K. What matters to patients? A systematic review of preferences for medication-associated outcomes in mental disorders. BMJ Open. 2015;5(4):e007848-e007848.
