# Supplementary material for: Patient-based benefit-risk assessment of medicines: development, refinement, and validation of a content search strategy to retrieve relevant studies
Source: J Med Libr Assoc. 2022 Apr 1;110(2):185–204. doi: 10.5195/jmla.2022.1306 (PMC9014953; doi:10.5195/jmla.2022.1306)
Supplement: Supplementary file 4 — Appendix 4: Articles included in two systematic reviews on patient preferences for attributes of disease-modifying drugs in multiple sclerosis [file jmla-110-2-185-s04.pdf]

Appendix 4: Articles included in two systematic reviews on patient preferences for attributes of disease-modifying drugs in multiple sclerosis

Table 1: Articles included in the systematic review on multiple sclerosis patients' understanding and preferences for risks and benefits of disease-modifying drugs [1]

**Reference**	**Year of publication**
**Articles included in the systematic review and published before August 2010**	
Mohr et al. [2]	1996
Miller et al. [3]	2001
Prosser et al. [4]	2002
Heesen et al. [5]	2003
Heesen et al. [6]	2004
Miller et al. [7]	2006
Kasper et al. [8]	2008
Johnson et al. [9]	2009
Vlahiotis et al. [10]	2010
**Articles included in the systematic review and published in and after August 2010**	
Visser et al. [11]	2011
de Seze et al. [12]	2012
Hofmann et al. [13]	2012
Miller et al. [14]	2012
Tur et al. [15]	2013
Abolfazli et al. [16]	2014
Köpke et al. [17]	2014
Syed et al. [18]	2014
Wilson et al. [19]	2014
Bruce et al. [20]	2015
Fox et al. [21]	2015
Wilson,et al. [22]	2015
Zimmer et al. [23]	2015

Table 2: Articles included in the systematic review of discrete-choice experiments and conjoint analysis studies in people with multiple sclerosis [24]

Reference	Year of publication
**Articles included in the systematic review and published before August 2010**	
Reed Johnson et al. [9]	2009
**Articles included in the systematic review and published in and after August 2010**	
Shingler et al. [25]	2013
Utz et al. [26]	2014
Wilson et al. [19]	2014
Wilson et al. [22]	2014
Rosato et al. [27]	2015
Wicks et al. [28]	2015
Garcia-Dominguez et al. [29]	2016
Kremer et al. [30]	2016
Lynd et al. [31]	2016
Poulos et al. [32]	2016
Poulos et al. [33]	2016
Arroyo et al. [34]	2017
Bottomley et al. [35]	2017
Carlin et al. [36]	2017
Sempere et al. [37]	2017

References

1. Reen GK, Silber E, Langdon DW. Multiple sclerosis patients' understanding and preferences for risks and benefits of disease-modifying drugs: a systematic review. J Neurol Sci. 2017;375:107–22.
2. Mohr DC, Goodkin DE, Likosky W, Gatto N, Neilley LK, Griffin C, Stiebling B. Therapeutic expectations of patients with multiple sclerosis upon initiating interferon beta-1b: relationship to adherence to treatment. Mult Scler. 1996;2(5):222–6.
3. Miller C, Jezewski MA. A phenomenologic assessment of relapsing MS patients' experiences during treatment with interferon beta-1a. J Neurosci Nurs. 2001;33(5):240–4.
4. Prosser LA, Kuntz KM, Bar-Or A, Weinstein MC. The relationship between risk attitude and treatment choice in patients with relapsing-remitting multiple sclerosis. Med Decis Making. 2002;22(6):506–13.
5. Heesen C, Kolbeck J, Gold SM, Schulz H, Schulz KH. Delivering the diagnosis of MS--results of a survey among patients and neurologists. Acta Neurol Scand. 2003;107(5):363–8.
6. Heesen C, Kasper J, Segal J, Köpke S, Mühlhauser I. Decisional role preferences, risk knowledge and information interests in patients with multiple sclerosis. Mult Scler. 2004;10(6):643–50.
7. Miller CE, Jezewski MA. Relapsing MS patients' experiences with glatiramer acetate treatment: a phenomenological study. J Neurosci Nurs. 2006;38(1):37–41.
8. Kasper J, Köpke S, Mühlhauser I, Nübling M, Heesen C. Informed shared decision making about immunotherapy for patients with multiple sclerosis (ISDIMS): a randomized controlled trial. Eur J Neurol. 2008;15(12):1345–52.
9. Johnson FR, Van Houtven G, Ozdemir S, Hass S, White J, Francis G, Miller DW, Phillips JT. Multiple sclerosis patients' benefit-risk preferences: serious adverse event risks versus treatment efficacy. J Neurol. 2009;256(4):554–62.
10. Vlahiotis A, Sedjo R, Cox ER, Burroughs TE, Rauchway A, Lich R. Gender differences in self-reported symptom awareness and perceived ability to manage therapy with disease-modifying medication among commercially insured multiple sclerosis patients. J Manag Care Pharm. 2010;16(3):206–16.
11. Visser LH, van der Zande A. Reasons patients give to use or not to use immunomodulating agents for multiple sclerosis. Eur J Neurol. 2011;18(11):1343–9.
12. de Seze J, Borgel F, Brudon F. Patient perceptions of multiple sclerosis and its treatment. Patient Prefer Adherence. 2012;6:263–73.
13. Hofmann A, Stellmann JP, Kasper J, Ufer F, Elias WG, Pauly I, Repenthin J, Rosenkranz T, Weber T, Köpke S, Heesen C, MS Network Hamburg. Long-term treatment risks in multiple sclerosis: risk knowledge and risk perception in a large cohort of mitoxantrone-treated patients. Mult Scler. 2013;19(7):920–5.
14. Miller CE, Karpinski M, Jezewski MA. Relapsing-remitting multiple sclerosis patients' experience with natalizumab: a phenomenological investigation. Int J MS Care. 2012;14(1):39–44.
15. Tur C, Tintoré M, Vidal-Jordana Á, Bichuetti D, Nieto González P, Arévalo MJ, Arrambide G, Anglada E, Galán I, Castilló J, Nos C, Río J, Martín MI, Comabella M, Sastre-Garriga J, Montalban X. Risk acceptance in multiple sclerosis patients on natalizumab treatment. PLoS One. 2013;8(12):e82796.
16. Abolfazli R, Elyasi A, Javadi MR, Gholami K, Torkamandi H, Amir-Shahkarami M, Etemadifar M, Nasr Z. Knowledge and attitude assessment of Iranian multiple sclerosis patients receiving interferon beta. Iran J Neurol. 2014;13(3):160–7.
17. Köpke S, Kern S, Ziemssen T, Berghoff M, Kleiter I, Marziniak M, Paul F, Vettorazzi E, Pöttgen J, Fischer K, Kasper J, Heesen C. Evidence-based patient information programme in early multiple sclerosis: a randomised controlled trial. J Neurol Neurosurg Psychiatry. 2014;85(4):411–8.

18. Syed M, Rog D, Parkes L, Shepherd GL. Patient expectations and experiences of multiple sclerosis interferon β-1a treatment: a longitudinal, observational study in routine UK clinical practice. Patient Prefer Adherence. 2014;8:247–55.
19. Wilson L, Loucks A, Bui C, Gipson G, Zhong L, Schwartzburg A, Crabtree E, Goodin D, Waubant E, McCulloch C. Patient centered decision making: use of conjoint analysis to determine risk-benefit trade-offs for preference sensitive treatment choices. J Neurol Sci. 2014;344(1-2):80–7.
20. Bruce JM, Bruce AS, Catley D, Lynch S, Goggin K, Reed D, Lim SL, Strober L, Glusman M, Ness AR, Jarmolowicz DP. Being kind to your future self: probability discounting of health decision-making. Ann Behav Med. 2016;50(2):297–309.
21. Fox RJ, Salter A, Alster JM, Dawson NV, Kattan MW, Miller D, Ramesh S, Tyry T, Wells BW, Cutter G. Risk tolerance to MS therapies: survey results from the NARCOMS registry. Mult Scler Relat Disord. 2015;4(3):241–9.
22. Wilson LS, Loucks A, Gipson G, Zhong L, Bui C, Miller E, Owen M, Pelletier D, Goodin D, Waubant E, McCulloch C. Patient preferences for attributes of multiple sclerosis disease-modifying therapies: development and results of a ratings-based conjoint analysis. Int J MS Care. 2015;17(2):74–82.
23. Zimmer A, Bläuer C, Coslovsky M, Kappos L, Derfuss T. Optimizing treatment initiation: Effects of a patient education program about fingolimod treatment on knowledge, self-efficacy and patient satisfaction. Mult Scler Relat Disord. 2015;4(5):444–50.
24. Webb EJD, Meads D, Eskyte I, King N, Dracup N, Chataway J, Ford HL, Marti J, Pavitt SH, Schmierer K, Manzano A. A systematic review of discrete-choice experiments and conjoint analysis studies in people with multiple sclerosis. Patient. 2018;11(4):391–402.
25. Shingler SL, Swinburn P, Ali S, Perard R, Lloyd AJ. A discrete choice experiment to determine patient preferences for injection devices in multiple sclerosis. J Med Econ. 2013;16(8):1036–42.
26. Utz KS, Hoog J, Wentrup A, Berg S, Lämmer A, Jainsch B, Waschbisch A, Lee DH, Linker RA, Schenk T. Patient preferences for disease-modifying drugs in multiple sclerosis therapy: a choice-based conjoint analysis. Ther Adv Neurol Disord. 2014;7(6):263–75.
27. Rosato R, Testa S, Oggero A, Molinengo G, Bertolotto A. Quality of life and patient preferences: identification of subgroups of multiple sclerosis patients. Qual Life Res. 2015;24(9):2173–82.
28. Wicks P, Brandes D, Park J, Liakhovitski D, Koudinova T, Sasane R. Preferred features of oral treatments and predictors of non-adherence: two web-based choice experiments in multiple sclerosis patients. Interact J Med Res. 2015;4(1):e6.
29. Garcia-Dominguez JM, Muñoz D, Comellas M, Gonzalbo I, Lizán L, Polanco Sánchez C. Patient preferences for treatment of multiple sclerosis with disease-modifying therapies: a discrete choice experiment. Patient Prefer Adherence. 2016;10:1945–56.
30. Kremer IE, Evers SM, Jongen PJ, van der Weijden T, van de Kolk I, Hiligsmann M. Identification and prioritization of important attributes of disease-modifying drugs in decision making among patients with multiple sclerosis: a nominal group technique and best-worst scaling. PLoS One. 2016;11(11):e0164862.
31. Lynd LD, Traboulsee A, Marra CA, Mittmann N, Evans C, Li KH, Carter M, Hategekimana C. Quantitative analysis of multiple sclerosis patients' preferences for drug treatment: a best-worst scaling study. Ther Adv Neurol Disord. 2016;9(4):287–96.
32. Poulos C, Kinter E, Yang JC, Bridges JF, Posner J, Gleißner E, Mühlbacher A, Kieseier B. A discrete-choice experiment to determine patient preferences for injectable multiple sclerosis treatments in Germany. Ther Adv Neurol Disord. 2016;9(2):95–104.
33. Poulos C, Kinter E, Yang JC, Bridges JF, Posner J, Reder AT. Patient preferences for injectable treatments for multiple sclerosis in the United States: a discrete-choice experiment. Patient. 2016;9(2):171–80.

34. Arroyo R, Sempere AP, Ruiz-Beato E, Prefasi D, Carreño A, Roset M, Maurino J. Conjoint analysis to understand preferences of patients with multiple sclerosis for disease-modifying therapy attributes in Spain: a cross-sectional observational study. BMJ Open. 2017;7(3):e014433.
35. Bottomley C, Lloyd A, Bennett G, Adlard N. A discrete choice experiment to determine UK patient preference for attributes of disease modifying treatments in Multiple Sclerosis. J Med Econ. 2017;20(8):863–70.
36. Carlin CS, Higuera L, Anderson S. Improving patient-centered care by assessing patient preferences for multiple sclerosis disease-modifying agents: a stated-choice experiment. Perm J. 2017;21:16–102.
37. Sempere AP, Vera-Lopez V, Gimenez-Martinez J, Ruiz-Beato E, Cuervo J, Maurino J. Using a multidimensional unfolding approach to assess multiple sclerosis patient preferences for disease-modifying therapy: a pilot study. Patient Prefer Adherence. 2017;11:995–9.
