# Supplementary material for: Patient-based benefit-risk assessment of medicines: development, refinement, and validation of a content search strategy to retrieve relevant studies
Source: J Med Libr Assoc. 2022 Apr 1;110(2):185–204. doi: 10.5195/jmla.2022.1306 (PMC9014953; doi:10.5195/jmla.2022.1306)
Supplement: Supplementary file 5 — Appendix 5: Testing the relative performance of the content search strategy by replicating a published systematic review [file jmla-110-2-185-s05.pdf]

Appendix 5: Testing the relative performance of the content search strategy by replicating a published systematic review

Table 1: Articles included in the systematic review on patient preferences for disease-modifying antirheumatic drug treatment in rheumatoid arthritis [1] and cited on PubMed, with corresponding keywords and MeSH terms

Reference	D*	ND**	Keywords / Free text words		MeSH terms	
			Patient-focused	Benefit-risk assessment of medicines	Patient-focused	Benefit-risk assessment of medicines
Fraenkel et al. [2]	x		- Patient perspective<br>- Treatment preferences	- Attribute<br>- Phenotypes	- Patient Preference*<br>- Choice Behavior<br>- Decision Making*	- Phenotype
Husni et al. [3]	x		- Patient preference	- Attributes<br>- Efficacy<br>- Safety<br>- Benefit-risk<br>- Treatment options	- Patient Preference*<br>- Choice Behavior<br>- Patient Participation*	- Risk Assessment<br>- Risk Factors<br>- Treatment Outcome
Alten et al. [4]	x		- Patient preferences	- Attributes<br>- Best and worst options		
Hazlewood et al. [5, 6]	x		- Treatment preferences<br>- Patient preference	- Benefits<br>- Harms<br>- Trade-offs<br>- Attributes<br>- Risk averse	- Patient Preference*<br>- Choice Behavior	- Risk Factors
Louder et al. [7]	x		- Patient preferences	- Attributes<br>- Part-worth utility		
Nolla et al. [8]	x		- Preferences	- Attributes<br>- Importance values<br>- Relative importance<br>- Most preferred<br>- Least preferred		
Fraenkel et al. [9]		x	- Patients' preferences<br>- Treatment preference	- Status quo<br>- Clinical inertia	- Patient Preference*<br>- Decision Making<br>- Patient Acceptance of Health Care / psychology*<br>- Patient Acceptance of Health Care / statistics & numerical data*	
Poulos et al. [10]	x			- Attributes<br>- Willingness<br>- Trade off<br>- Efficacy<br>- Risk	- Patient Preference / statistics & numerical data*	

Augustovski et al. [11]	x		- Patient preferences	- Attributes<br>- Trade-offs<br>- Relative importance	- Choice Behavior<br>- Patient Preference / psychology<br>- Patient Preference / statistics & numerical data*	
Constantinescu et al. [12, 13]	x		- Patient preferences<br>- Treatment preferences	- Risks<br>- Benefits<br>- Ratio of importance<br>- Greatest risk<br>- Risk aversion	Decision Making<br>Health Knowledge, Attitudes, Practice	- Risk Assessment
Ozdemir et al. [14]	x			- Attributes<br>- Treatment alternatives<br>- Willingness to pay	- Choice Behavior*<br>- Patient Acceptance of Health Care*	- Cost-Benefit Analysis
Skjoldborg et al. [15]	x			- Willingness to pay	- Choice Behavior*<br>- Patient Acceptance of Health Care / psychology	
Fraenkel et al. [16]	x		- Patient preferences<br>- Treatment preferences<br>- Patient's perspective	- Trade-off<br>- Treatment options<br>- Maximum benefits<br>- Adverse events	- Patient Satisfaction*	- Risk
Chiou et al. [17]	x		- Preference	- Tradeoff<br>- Adverse events<br>- Health states		- Treatment Outcome
Suarez-Almazor and Conner-Spady [18]	x		- Preferences	- Tradeoff<br>- Health states	- Patient Satisfaction / economics*	- Cost-Benefit Analysis
Tuominen et al. [19]	x			- Value<br>- Willingness-to-pay	- Patient Acceptance of Health Care	
Slothuus et al. [20, 21]		x		- Willingness<br>- Cost-benefit<br>- Trade-off	- Patient Acceptance of Health Care	- Cost-Benefit Analysis
Fraenkel et al. [22, 23]	x			- Willingness<br>- Risk<br>- Adverse effects<br>- Risk adversity<br>- Trade-offs	- Health Knowledge, Attitudes, Practice*<br>- Patient Acceptance of Health Care / psychology*<br>- Decision Making<br>- Patient Acceptance of Health Care<br>- Patient Satisfaction*	- Risk-Taking*<br>- Risk Assessment
Ho et al. [24]		x	- Patients' attitudes	- Risk<br>- Acceptable risk<br>- Risk: benefit ratio	- Attitude to Health*<br>- Patient Satisfaction	- Risk Assessment<br>- Risk-Taking*

O'Brien et al. [25]	x			- Willingness<br>- Risk<br>- Risk-benefit preference	- Choice Behavior<br>- Patient Acceptance of Health Care*<br>- Attitude to Health	- Risk-Taking*
Bacalao et al. [26]	x		- Patient preferences	- Domain<br>- Most important<br>- Values<br>- Willingness	- Patient Participation*	
van Tuyl et al. [27]	x		- Patient perspective	- Domain<br>- Most important aspect	- Attitude to Health*	- Patient Outcome Assessment
Buitinga et al. [28]	x			- Trade-off<br>- Worst-case	- Patient Preference*	
Sanderson et al. [29]	x		- Patient perspective	- Priorities<br>- Efficacy<br>- Effectiveness<br>- Treatment outcomes	- Attitude to Health<br>- Patient Satisfaction*	- Treatment Outcome
Da Silva et al. [30]		x	- Patients' needs and preferences	- Priority		- Health Priorities*
Heiberg et al. [31]	x		- Patient preferences	- Benefit<br>- Efficacy	- Patient Satisfaction*	
Desplats et al. [32]	x		- Patients' preferences	- Route of administration<br>- Adverse events	- Patient Preference / statistics & numerical data	
Bolge et al. [33]	x		- Preference<br>- Perspective<br>- Openness	- Attribute<br>- Route of administration		
Navarro-Millan et al. [34]	x		- Patient preferences	- Effectiveness		
Huynh et al. [35]	x		- Preferences of patients	- Route and frequency of administration<br>- Adverse effects<br>- Safety		
Scarpato et al. [36]	x		- Patient preferences	- Route of administration	- Patient Preference*<br>- Choice Behavior	
Martin et al. [37]	x			- Efficacy<br>- Treatment outcomes<br>- Beliefs about medications	- Decision Making*	
Van Overbeeke et al. [38]	x			- Efficacy<br>- Side effects<br>- Suitability	- Patient Preference<br>- Health Knowledge, Attitudes, Practice*	
Fraenkel et al. [39]	x				- Health Knowledge, Attitudes, Practice<br>- Patient Participation*	- Risk Assessment<br>- Risk Factors
Goekoop - Ruiterman et al. [40]		x	- Patient preferences	- Aversion		- Treatment Outcome

			- Treatment preferences			

D: Detected by the content search strategy combined, using AND, with a search syntax relevant to rheumatoid arthritis and its treatment: Rheumatoid arthritis OR RA OR Rheumatic diseases OR disease-modifying antirheumatic drugs OR DMARDs OR antirheumatic agents OR Arthritis, Rheumatoid / drug therapy*[MeSH] OR Arthritis, Rheumatoid / therapy*[MeSH] OR Antirheumatic Agents / therapeutic use*[MeSH] OR Biological Products / therapeutic use*[MeSH]

ND: Not detected by the content search strategy combined, using AND, with a search syntax relevant to rheumatoid arthritis and its treatment: Rheumatoid arthritis OR RA OR Rheumatic diseases OR disease-modifying antirheumatic drugs OR DMARDs OR antirheumatic agents OR Arthritis, Rheumatoid / drug therapy*[MeSH] OR Arthritis, Rheumatoid / therapy*[MeSH] OR Antirheumatic Agents / therapeutic use*[MeSH] OR Biological Products / therapeutic use*[MeSH]

Table 2: Quality assessment of included studies in the systematic review on patient preferences for disease-modifying antirheumatic drug treatment in rheumatoid arthritis [1], based on a practical tool developed to critically assess patient preference studies across methodologies [41]

Reference	External validity	Quality of construct representation	Minimization of the risk of construct-irrelevant variance	Quality of reporting and analysis	Other aspects that strengthen or weaken the study	Overall quality
Fraenkel et al. [2]	Medium	Medium	Moderate	Medium	No difference	Medium
Husni et al. [3]	Medium	Medium	Moderate	High	No difference	Medium
Alten et al. [4]	High	High	Moderate	High	No difference	High
Hazlewood et al. [5, 6]	High	Medium	High	High	Strengthen	High
Louder et al. [7]	Low	Medium	High	High	Weaken	Low
Nolla et al. [8]	Medium	Medium	Moderate	High	No difference	Medium
Fraenkel et al. [9]	High	Medium	Moderate	High	No difference	High
Poulos et al. [10]	Low	Medium	High	High	No difference	Medium
Augustovski et al. [11]	High	Medium	High	High	Strengthen	High
Constantinescu et al. [12, 13]	High	Medium	Moderate	High	No difference	Medium
Ozdemir et al. [14]	Medium	Low	High	High	No difference	Medium
Skjoldborg et al. [15]	Medium	Low	Low	High	Strengthen	Medium
Fraenkel et al. [16]	Medium	Medium	Moderate	High	No difference	Medium
Chiou et al. [17]	Medium	Medium	Moderate	High	No difference	Medium
Suarez-Almazor and Conner-Spady [18]	Medium	High	High	High	Strengthen	High
Tuominen et al. [19]	High	Medium	Moderate	Medium	Weaken	Medium
Slothuus et al. [20, 21]	Medium	Medium	High	High	Strengthen	High
Fraenkel et al. [22, 23]	Medium	Medium	Moderate	High	No difference	Medium
Ho et al. [24]	Medium	Low	Low	Low	Weaken	Low
O'Brien et al. [25]	Low	Medium	Moderate	High	No difference	Low
Bacalao et al. [26]	Medium	High	High	High	No difference	High
van Tuyl et al. [27]	High	High	High	High	No difference	High
Buitinga et al. [28]	Medium	High	High	High	No difference	High
Sanderson et al. [29]	High	High	High	High	No difference	High

Da Silva et al. [30]	High	High	High	High	No difference	High
Heiberg et al. [31]	Medium	High	High	High	No difference	High
Desplats et al. [32]	High	Medium	Moderate	High	No difference	Medium
Bolge et al. [33]	Low	Medium	Low	High	No difference	Low
Navarro-Millan et al. [34]	Medium	High	High	High	No difference	Medium
Huynh et al. [35]	Medium	High	High	High	No difference	Medium
Scarpato et al. [36]	High	High	High	High	No difference	High
Martin et al. [37]	Medium	Medium	Moderate	Medium	No difference	Medium
Van Overbeeke et al. [38]	Low	High	Moderate	High	No difference	Medium
Fraenkel et al. [39]	Low	High	High	High	No difference	Medium
Goekoop - Ruiterman et al. [40]	High	Medium	Moderate	High	No difference	Medium

References

1. Durand C, Eldoma M, Marshall DA, Bansback N, Hazlewood GS. Patient preferences for disease-modifying antirheumatic drug treatment in rheumatoid arthritis: a systematic review. J Rheumatol. 2020;47(2):176–87.
2. Fraenkel L, Nowell WB, Michel G, Wiedmeyer C. Preference phenotypes to facilitate shared decision-making in rheumatoid arthritis. Ann Rheum Dis. 2018;77(5):678–83.
3. Husni ME, Betts KA, Griffith J, Song Y, Ganguli A. Benefit-risk trade-offs for treatment decisions in moderate-to-severe rheumatoid arthritis: focus on the patient perspective. Rheumatol Int. 2017;37(9):1423–34.
4. Alten R, Krüger K, Rellecke J, Schiffner-Rohe J, Behmer O, Schiffhorst G, Nolting HD. Examining patient preferences in the treatment of rheumatoid arthritis using a discrete-choice approach. Patient Prefer Adherence. 2016;10:2217–28.
5. Hazlewood GS, Bombardier C, Tomlinson G, Thorne C, Bykerk VP, Thompson A, Tin D, Marshall DA. Treatment preferences of patients with early rheumatoid arthritis: a discrete-choice experiment. Rheumatology (Oxford). 2016;55(11):1959–68.
6. Hazlewood GS, Bombardier C, Tomlinson G, Marshall D. A Bayesian model that jointly considers comparative effectiveness research and patients' preferences may help inform GRADE recommendations: an application to rheumatoid arthritis treatment recommendations. J Clin Epidemiol. 2018;93:56–65.
7. Louder AM, Singh A, Saverno K, Cappelleri JC, Aten AJ, Koenig AS, Pasquale MK. Patient preferences regarding rheumatoid arthritis therapies: a conjoint analysis. Am Health Drug Benefits. 2016;9(2):84–93.
8. Nolla JM, Rodríguez M, Martin-Mola E, Raya E, Ibero I, Nocea G, Aragon B, Lizán L, Prades M. Patients' and rheumatologists' preferences for the attributes of biological agents used in the treatment of rheumatic diseases in Spain. Patient Prefer Adherence. 2016;10:1101–13.
9. Fraenkel L, Cunningham M, Peters E. Subjective numeracy and preference to stay with the status quo. Med Decis Making. 2015;35(1):6–11.
10. Poulos C, Hauber AB, González JM, Turpcu A. Patients' willingness to trade off between the duration and frequency of rheumatoid arthritis treatments. Arthritis Care Res (Hoboken). 2014;66(7):1008–15.
11. Augustovski F, Beratarrechea A, Irazola V, Rubinstein F, Tesolin P, Gonzalez J, Lencina V, Scolnik M, Waimann C, Navarta D, Citera G, Soriano ER. Patient preferences for biologic agents in rheumatoid arthritis: a discrete-choice experiment. Value Health. 2013;16(2):385-93.
12. Constantinescu F, Goucher S, Weinstein A, Smith W, Fraenkel L. Understanding why rheumatoid arthritis patient treatment preferences differ by race. Arthritis Rheum. 2009;61(4):413–8.
13. Constantinescu F, Goucher S, Weinstein A, Fraenkel L. Racial disparities in treatment preferences for rheumatoid arthritis. Med Care. 2009;47(3):350–5.
14. Ozdemir S, Johnson FR, Hauber AB. Hypothetical bias, cheap talk, and stated willingness to pay for health care. J Health Econ. 2009;28(4):894–901.
15. Skjoldborg US, Lauridsen J, Junker P. Reliability of the discrete choice experiment at the input and output level in patients with rheumatoid arthritis. Value Health. 2009;12(1):153–8.
16. Fraenkel L, Bogardus ST, Concato J, Felson DT, Wittink DR. Patient preferences for treatment of rheumatoid arthritis. Ann Rheum Dis. 2004;63(11):1372–8.
17. Chiou CF, Weisman M, Sherbourne CD, Reyes C, Dylan M, Ofman J, Wallace DJ, Mizutani W, Suarez-Almazor ME. Measuring preference weights for American college of rheumatology response criteria for patients with rheumatoid arthritis. J Rheumatol. 2005;32(12):2326–9.
18. Suarez-Almazor ME, Conner-Spady B. Rating of arthritis health states by patients, physicians, and the general public. Implications for cost-utility analyses. J Rheumatol. 2001;28(3):648–56.
19. Tuominen R, Tuominen S, Möttönen T. How much is a reduction in morning stiffness worth to patients with rheumatoid arthritis? Scand J Rheumatol Suppl. 2011;125:12–16.

20. Slothuus U, Brooks RG. Willingness to pay in arthritis: a Danish contribution. Rheumatology (Oxford). 2000;39(7):791–9.
21. Slothuus U, Larsen ML, Junker P. Willingness to pay for arthritis symptom alleviation. Comparison of closed-ended questions with and without follow-up. Int J Technol Assess Health Care. 2000;16(1):60–72.
22. Fraenkel L, Bogardus S, Concato J, Felson D. Unwillingness of rheumatoid arthritis patients to risk adverse effects. Rheumatology (Oxford). 2002;41(3):253–61.
23. Fraenkel L, Bogardus S, Concato J, Felson D. Risk communication in rheumatoid arthritis. J Rheumatol. 2003;30(3):443–8.
24. Ho M, Lavery B, Pullar T. The risk of treatment. A study of rheumatoid arthritis patients' attitudes. Br J Rheumatol. 1998;37(4):459–60.
25. O'Brien BJ, Elswood J, Calin A. Willingness to accept risk in the treatment of rheumatic disease. J Epidemiol Community Health. 1990;44(3):249–52.
26. Bacalao EJ, Greene GJ, Beaumont JL, Eisenstein A, Muftic A, Mandelin AM, Cella D, Ruderman EM. Standardizing and personalizing the treat to target (T2T) approach for rheumatoid arthritis using the Patient-Reported Outcomes Measurement Information System (PROMIS): baseline findings on patient-centered treatment priorities. Clin Rheumatol. 2017;36(8):1729–36.
27. van Tuyl LH, Sadlonova M, Hewlett S, Davis B, Flurey C, Goel N, Gossec L, Brahe CH, Hill CL, Hoogland W, Kirwan J, Hetland ML, van Schaardenburg D, Smolen JS, Stamm T, Voshaar M, Wells GA, Boers M. The patient perspective on absence of disease activity in rheumatoid arthritis: a survey to identify key domains of patient-perceived remission. Ann Rheum Dis. 2017;76(5):855–61.
28. Buitinga L, Braakman-Jansen LM, Taal E, van de Laar MA. Worst-case future scenarios of patients with rheumatoid arthritis: a cross-sectional study. Rheumatology (Oxford). 2012;51(11):2027–33.
29. Sanderson T, Morris M, Calnan M, Richards P, Hewlett S. Patient perspective of measuring treatment efficacy: the rheumatoid arthritis patient priorities for pharmacologic interventions outcomes. Arthritis Care Res (Hoboken). 2010;62(5):647–56.
30. da Silva JA, Ramiro S, Pedro S, Rodrigues A, Vasconcelos JC, Benito-Garcia E. Patients- and physicians- priorities for improvement. The case of rheumatic diseases. Acta Reumatol Port. 2010;35(2):192–9.
31. Heiberg T, Kvien TK. Preferences for improved health examined in 1,024 patients with rheumatoid arthritis: pain has highest priority. Arthritis Rheum. 2002;47(4):391–7.
32. Desplats M, Pascart T, Jelin G, Norberciak L, Philippe P, Houvenagel E, Goeb V, Flipo RM. Are abatacept and tocilizumab intravenous users willing to switch for the subcutaneous route of administration? A questionnaire-based study. Clin Rheumatol. 2017;36(6):1395–400.
33. Bolge SC, Goren A, Brown D, Ginsberg S, Allen I. Openness to and preference for attributes of biologic therapy prior to initiation among patients with rheumatoid arthritis: patient and rheumatologist perspectives and implications for decision making. Patient Prefer Adherence. 2016;10:1079–90.
34. Navarro-Millán I, Herrinton LJ, Chen L, Harrold L, Liu L, Curtis JR. Comparative effectiveness of etanercept and adalimumab in patient reported outcomes and injection-related tolerability. PLoS One. 2016;11(3):e0149781.
35. Huynh TK, Ostergaard A, Egsmose C, Madsen OR. Preferences of patients and health professionals for route and frequency of administration of biologic agents in the treatment of rheumatoid arthritis. Patient Prefer Adherence. 2014;8:93–9.
36. Scarpato S, Antivalle M, Favalli EG, Nacci F, Frigelli S, Bartoli F, Bazzichi L, Minisola G, Cerinic MM, RIVIERA co-authors. Patient preferences in the choice of anti-TNF therapies in rheumatoid arthritis. Results from a questionnaire survey (RIVIERA study). Rheumatology (Oxford). 2010;49(2):289–94.

37. Martin RW, Enck RD, Tellinghuisen DJ, Eggebeen AT, Birmingham JD, Head AJ. Comparison of the effects of a pharmaceutical industry decision guide and decision aids on patient choice to intensify therapy in rheumatoid arthritis. Med Decis Making. 2017;37(5):577–88.
38. van Overbeeke E, De Beleyr B, de Hoon J, Westhovens R, Huys I. Perception of originator biologics and biosimilars: a survey among belgian rheumatoid arthritis patients and rheumatologists. BioDrugs. 2017;31(5):447–59.
39. Fraenkel L, Miller AS, Clayton K, Crow-Hercher R, Hazel S, Johnson B, Rott L, White W, Wiedmeyer C, Montori VM, Singh JA, Nowell WB. When patients write the guidelines: patient panel recommendations for the treatment of rheumatoid arthritis. Arthritis Care Res (Hoboken). 2016;68(1):26–35.
40. Goekoop-Ruiterman YP, de Vries-Bouwstra JK, Allaart CF, Kerstens PJ, Grillet BA, de Jager MH, Han KH, Speyer I, van der Lubbe PAHM, Seys PES, Breedveld FC, Dijkmans BAC. Patient preferences for treatment: report from a randomised comparison of treatment strategies in early rheumatoid arthritis (BeSt trial). Ann Rheum Dis. 2007;66(9):1227–32.
41. Eiring Ø, Landmark BF, Aas E, Salkeld G, Nylenna M, Nytrøen K. What matters to patients? A systematic review of preferences for medication-associated outcomes in mental disorders. BMJ Open. 2015;5(4):e007848-e007848.
