# Supplementary material for: Patient-based benefit-risk assessment of medicines: development, refinement, and validation of a content search strategy to retrieve relevant studies
Source: J Med Libr Assoc. 2022 Apr 1;110(2):185–204. doi: 10.5195/jmla.2022.1306 (PMC9014953; doi:10.5195/jmla.2022.1306)
Supplement: Supplementary file 6 — Appendix 6: Reproducible search strategies on patient-based benefit-risk assessment of medicines in PubMed [file jmla-110-2-185-s06.pdf]

Appendix 6. Reproducible search strategies on patient-based benefit-risk assessment of medicines in PubMed

Reproducible search on patient-based benefit-risk assessment of medicines in PubMed:

(((((((((((((patient preference[MeSH Terms]) OR (Patient Preference / psychology[MeSH Subheading])) OR (Patient Preference / statistics & numerical data[MeSH Subheading])) OR (Patient Preference*[Title/Abstract])) OR (Patients preference*[Title/Abstract])) OR (Patient perception[Title/Abstract])) OR (Stated preference*[Title/Abstract])) OR (Treatment preference[Title/Abstract])) OR (Preference[Title/Abstract])) OR (Perspective[Title/Abstract])) OR (choice behavior[MeSH Terms])) OR (decision making[MeSH Terms])) OR (health knowledge, attitudes, practice[MeSH Terms])) AND ((((((((((((((((((((((((attribute*[Title/Abstract]) OR (benefit*[Title/Abstract])) OR (benefit-risk)) OR (risk tolerance)) OR (trade-off*)) OR (tradeoff*)) OR (efficacy[Title/Abstract])) OR (safety[Title/Abstract])) OR (side effect*[Title/Abstract])) OR (adverse event*[Title/Abstract])) OR (effectiveness[Title/Abstract])) OR (frequency[Title/Abstract])) OR (accepta*[Title/Abstract])) OR (Maximum acceptable risk)) OR (Minimum acceptable efficacy)) OR (Preferred treatment option[Title/Abstract])) OR (Patient-reported outcome*[Title/Abstract])) OR (Relative importance[Title/Abstract])) OR (most preferred[Title/Abstract])) OR (least preferred[Title/Abstract])) OR (willingness[Title/Abstract])) OR (risk assessment[MeSH Terms])) OR (benefit risk assessment[MeSH Terms])) OR (Drug-related side effects and adverse reactions/psychology[MeSH Terms]))

Reproducible extended search on patient-based benefit-risk assessment of medicines in PubMed:

((((((((((((((((((patient preference[MeSH Terms]) OR (patient preference/ psychology[MeSH Subheading])) OR (patient preference/ statistics & numerical data[MeSH Subheading])) OR (patient preference*[Title/Abstract])) OR (patients preference[Title/Abstract])) OR (perception[Title/Abstract])) OR (stated preference*[Title/Abstract])) OR (treatment preference[Title/Abstract])) OR (treatment satisfaction[Title/Abstract])) OR (willingness[Title/Abstract])) OR (willingness to pay)) OR (patient concerns[Title/Abstract])) OR (choice behavior[MeSH Terms])) OR (decision making[MeSH Terms])) OR (health knowledge, attitudes, practice[MeSH Terms])) OR (attitude to health[MeSH Terms])) OR (patient acceptance of health care[MeSH Terms])) OR (patient acceptance of health care/psychology[MeSH Terms])) AND ((attribute*[Title/Abstract]) OR (benefit*[Title/Abstract])) OR (benefit-risk)) OR (risk tolerance)) OR (risk awareness)) OR (risk perception)) OR (Trade-off*)) OR (tradeoff*)) OR (efficacy[Title/Abstract])) OR (safety[Title/Abstract])) OR (side effect*[Title/Abstract])) OR (adverse event*[Title/Abstract])) OR (probability of occurence[Title/Abstract])) OR (effectiveness[Title/Abstract])) OR (frequency[Title/Abstract])) OR (value[Title/Abstract])) OR (utility[Title/Abstract])) OR

(disutility[Title/Abstract])) OR (accepta*[Title/Abstract])) OR (maximum acceptable risk)) OR (minimum acceptable efficacy)) OR (acceptable regimen[Title/Abstract])) OR (preferred treatment option[Title/Abstract])) OR (patient-reported outcome*[Title/Abstract])) OR (relative importance[Title/Abstract])) OR (most preferred[Title/Abstract])) OR (least preferred[Title/Abstract])) OR (medication belie*[Title/Abstract])) OR (discontinuation[Title/Abstract])) OR (standard gamble[Title/Abstract])) OR (discrete choice experiment[Title/Abstract])) OR (conjoint analysis[Title/Abstract])) OR (benefit risk assessment[MeSH Terms])) OR (risk assessment[MeSH Terms])) OR (risk reduction behavior[MeSH Terms])) OR (Drug-related side effects and adverse reactions/psychology[MeSH Terms])) OR (risk[MeSH Terms])) OR (treatment outcome[MeSH Terms])) OR (drug administration routes[MeSH Terms])) OR (drug administration schedule[MeSH Terms])) OR (Outcome and Process Assessment, Health Care[MeSH Terms])) OR (Outcome Assessment, Health Care / methods[MeSH Terms]))
